# Multiple Contrast Tests for Count Data: Small Sample Approximations and Their Limitations

**DOI:** 10.1002/bimj.70098

**Published:** 2025-12-07

**Authors:** Mareen Pigorsch, Ludwig A. Hothorn, Frank Konietschke

**Affiliations:** ^1^ Charité – Universitätsmedizin Berlin Institute of Biometry and Clinical Epidemiology Berlin Germany; ^2^ retired from Leibniz University Hannover Lauenau Germany

## Abstract

Although count data are collected in many experiments, their analysis remains challenging, especially in small sample sizes. Until now, linear or generalized linear models in Poisson or Negative Binomial distributional families have often been used. However, these data frequently show signs of over‐, underdispersion, or even zero‐inflation, casting doubt on these distributional assumptions and leading to inaccurate test results. Since their distributions are usually skewed, data transformations (e.g., log‐transformation) are not unusual. This underscores the need for statistical methods not to hinge on specific distributional assumptions. We delve into multiple contrast tests that allow general contrasts (e.g., many‐to‐one or all‐pairs comparisons) to analyze count data in multi‐arm trials. The methods vary in their effect and variance estimation, as well as in approximating the joint distribution of multiple test statistics, including frequently used methods such as linear and generalized linear models, and data transformations. An extensive simulation study demonstrates that a resampling version effectively controls the Type I error rate in various situations, while also highlighting the method's limitations, including overly liberal Type I error rates. Some standard methods, which have inflated Type I error rates, further underscore the need for alternative approaches. Real data applications further emphasize the applicability of these methods.

## 1 Introduction

Count data frequently occur in clinical trials and fields like biology and ecotoxicology (Szöcs and Schäfer 2015; St‐Pierre et al. 2018). Typical examples from the clinical context include the number of hospitalizations, doctor visits, or the length of hospital stay in days. This kind of data is discrete and often right‐skewed. In addition, overdispersion, that is, the variance exceeds the mean, underdispersion, and zero‐inflation can occur. These distributional properties challenge statistical inference when estimating treatment effects and other parameters, such as the dispersion parameter, testing null hypotheses, or computing confidence intervals, especially in small sample sizes. Ignoring the dispersion ends in standard errors (SEs) of the effect estimators that are too small or too large. Typically, dispersion is modeled as a global effect and is equal across the treatment groups; here, we allow for group‐wise dispersions. Linear and generalized linear models (GLMs), for example, assuming Poisson or Negative Binomial distribution, are often used to analyze count data. Szöcs and Schäfer (2015) recommend that counts be analyzed using quasi‐Poisson models, which can account for over‐ and underdispersion in a unified way, respectively. However, specifying the explicit underlying distributional family to be used within the GLM is impossible when sample sizes are small. Therefore, the procedures usually tend to behave either liberally or conservatively, over‐ or under‐rejecting the null hypothesis, thus yielding false positive or false negative conclusions. To overcome distributional misspecifications, normalizing (symmetrizing) their distribution toward a normal distribution with appropriate data transformations (e.g., the log‐transformation) is a common strategy in statistical practice and has been discussed previously. While O'Hara and Kotze (2010) recommend not using log‐transformation to analyze count data but instead using Poisson or Negative Binomial GLM, Ives (2015) advises to log‐transform the data, followed by using a linear model. Warton (2018) argues that the transformation cannot stabilize variances for small counts.

However, all these strategies and the inferential methods rely on (strict) distributional assumptions that the data may not fulfill; hence, the procedures may behave liberally, over‐reject the null hypothesis, and end in false positive conclusions under various circumstances. To overcome these drawbacks, we will first introduce a general statistical model for count data that does not rely on an explicit distributional assumption. Second, we will introduce a nonparametric bootstrap method that allows for heteroscedasticity and group‐specific dispersion as a small‐sample‐size approximation. Compared with its competitors, the bootstrap test does not rely on distributional assumptions. Multi‐arm trials are considered, where simultaneous comparisons, for example, multiple comparisons to control or testing Grand‐Mean contrasts, are of interest and will be implemented using multiple contrast test procedures (MCTPs) (Bretz et al. 2001; Konietschke et al. 2012). Compared to global test procedures, such as quadratic forms used within the analysis of variance (ANOVA) framework, MCTPs are more flexible, allowing user‐specific contrasts. While global tests can only provide information on whether any groups differ in the case of a globally significant outcome, MCTPs provide local *p*‐values (adjusted for multiplicity) and simultaneous confidence intervals that reveal the guilty levels in the case of a significant result. Therefore, they are often prefered, especially in preclinical stages and toxicology. Note that Kruppa and Hothorn (2021) also studied MCTPs for equally overdispersed clustered count data in a comprehensive simulation study. While statistical modeling and data generation were crucial questions, their focus was different: We aim to investigate the impacts of (a) model misspecification on various MCTPs in small sample sizes and (b) data transformations on the behavior of MCTPs. In addition, we do not only employ the impact of the usual Poisson and Negative Binomial distributions but also of another less prominent candidate, the Conway–Maxwell–Poisson distribution, on existing and novel test procedures.

The paper is organized as follows: In Section 2, we present the statistical model, multiple null hypotheses and contrasts, the point estimates, and their asymptotic distribution. Section 3 introduces multiple contrast tests, while Section 4 overviews the examined methods, including a nonparametric bootstrap test. The results of the comprehensive simulation study are presented in Section 5. An illustrative real‐data application is provided in Section 6. Finally, our findings are discussed in Section 7. Additional results of the simulation study can be found in the Supporting Information.

## 2 Statistical Model and Point Estimates

We consider a general one‐way count data design comprising a independent groups of $n_{i}$ independent subjects (units) each, where the index i=1,…,a denotes the respective group. The total sample size is given by $N=\sum_{i=1}^{a}n_{i}$. In standard cases, count data are assumed to follow specific distributions, such as a Poisson or Negative Binomial model, thereby allowing for overdispersion. These specifications limit the model's flexibility and applicability. In small sample sizes, particularly, validating a specific statistical model is nearly impossible, often resulting in false conclusions due to model misspecification. We, therefore, model the counts by independent and identically distributed random variables

(1) $$X_{ik}∼F_{i}\left(\lambda_{i},\sigma_{i}^{2}\right),i=1,\text{…},a;\;k=1,\text{…},n_{i},$$

and a general (arbitrary) distribution $F_{i}\left(\lambda_{i},\sigma_{i}^{2}\right)$, characterized by a group‐specific count rate $\lambda_{i}$ and variance $\sigma_{i}^{2}$, respectively. We do not restrict $\sigma_{i}^{2}$ to depend on $\lambda_{i}$; both can be unrelated, so Model 1 covers under‐ and overdispersed count data as special cases. Note that the group‐specific variances $\sigma_{i}^{2}$ involve group‐specific dispersions as special cases; we do not interpret/restrict dispersion as a global effect/impact. The model parameters, however, are unknown and must be estimated from the data in practical applications. Unbiased estimators of $\lambda_{i}$ and $\sigma_{i}^{2}$ are given by their empirical counterparts

$${\hat{\lambda }}_{i}=\frac{1}{n_{i}}\sum_{k=1}^{n_{i}}X_{\mathrm{ik}}\;\text{and}\;{\hat{\sigma }}_{i}^{2}=\frac{1}{n_{i}-1}\sum_{k=1}^{n_{i}}{\left(X_{\mathrm{ik}}-{\hat{\lambda }}_{i}\right)}^{2},\;i=1,\cdots ,a,$$

respectively. Most existing procedures estimate model parameters under specific data distributional assumptions using maximum likelihood methods, such as generalized linear models. In the general situation considered here, empirical likelihood estimators may be an option; however, stable estimation and asymptotic theory require fairly large sample sizes. We, therefore, stick to the introduced empirical quantities and discuss variance‐stabilizing functions later.

Remark 2.1 In some situations, interpreting the dispersion as a global (data) effect is necessary. In these situations, we recommend estimating dispersion from an appropriate model, for example, using maximum likelihood methods or moment‐based estimators.

For the derivation of asymptotic results and interpretability, the following mild assumptions on the variances and sample sizes $n_{i}$ are necessary:

**A1**: Each variance $\sigma_{i}^{2}>0$, which excludes one‐point and other degenerate distributions.
**A2**: The sample sizes are also asymptotically not too unbalanced, that is, N→∞ such that
$\frac{N}{n_{i}}\;\leq \;N_{0}\;<\;\infty$.

In the general statistical model considered here, making inferences in its parameters, especially in the count rates $\lambda ={\left(\lambda_{1},\text{…},\lambda_{a}\right)}^{\prime }$ is of major interest. The research questions are manifold but specific, and hence, the alternative hypotheses are characterized by a specific pattern in λ. We will provide details and discuss examples in the next subsection. Before, we add a remark about a generalized version of Model 1.

Remark 2.2 Sometimes, counts are observed within specific and individual time frames. In these cases, the statistical model generalizes to

(2) $$X_{ik}∼F_{ik}\left(t_{ik}\lambda_{i},\sigma_{ik}^{2}\right),i=1,\text{…},a;\;k=1,\text{…},n_{i},$$

where $t_{ik}$ is the offset parameter and denotes a known and individually fixed period. In these, the distributions $F_{ik}$ become subject‐specific and completely heteroscedastic, meaning that each variable has (or may have) a different variance $\sigma_{ik}^{2}>0$. For ease of reading, we do not consider Model 2 in the main part of the paper, but instead generalize the results in Section 7.

### 2.1 Hypotheses

In several samples, the null and alternative hypotheses are usually formulated as

$$\begin{array}{c} H_{0}:\lambda_{1}=\cdots =\lambda_{a}\;\text{versus}\;H_{1}:\lambda_{i}\neq \lambda_{j};\;\;i\neq j. \end{array}$$

In other words, the above means that if an appropriate procedure rejects $H_{0}$, any of the a competitors causes a significant difference. In statistical practice, however, the specific identification of the guilty groups is of paramount importance, and hence, the formulation of $H_{1}$ above is too imprecise. The aim of many trials and experiments, especially in preclinical and early stages, is to detect a certain pattern of differences between $\lambda_{1},\text{…},\lambda_{a}$, summarized in a q×a contrast matrix C. Each of the q row vectors $c_{\ell }^{\prime }=\left(c_{\ell 1},\text{…},c_{\ell a}\right)$ constitutes one specific contrast. For example, multiple comparisons to a control (many‐to‐one) (Dunnett 1955) can be expressed as follows, exemplary for four groups,

$$H_{0}:\left\{\begin{array}{c} \lambda_{2}=\lambda_{1}\\ \lambda_{3}=\lambda_{1}\\ \lambda_{4}=\lambda_{1} \end{array}\right.\Leftrightarrow H_{0}:C\lambda =\left(\begin{array}{cccc} -1 & 1 & 0 & 0\\ -1 & 0 & 1 & 0\\ -1 & 0 & 0 & 1 \end{array}\right)\left(\begin{array}{c} \lambda_{1}\\ \lambda_{2}\\ \lambda_{3}\\ \lambda_{4} \end{array}\right)=0.$$

Tukey‐type all‐pairs (Tukey 1953) comparisons can be represented with

$$H_{0}:\left\{\begin{array}{c} \lambda_{2}=\lambda_{1}\\ \lambda_{3}=\lambda_{1}\\ \lambda_{4}=\lambda_{1}\\ \lambda_{3}=\lambda_{2}\\ \lambda_{4}=\lambda_{2}\\ \lambda_{4}=\lambda_{3} \end{array}\right.\Leftrightarrow H_{0}:C\lambda =\left(\begin{array}{cccc} -1 & 1 & 0 & 0\\ -1 & 0 & 1 & 0\\ -1 & 0 & 0 & 1\\ 0 & -1 & 1 & 0\\ 0 & -1 & 0 & 1\\ 0 & 0 & -1 & 1 \end{array}\right)\left(\begin{array}{c} \lambda_{1}\\ \lambda_{2}\\ \lambda_{3}\\ \lambda_{4} \end{array}\right)=0.$$

Furthermore, the Grand‐Mean (Pallmann and Hothorn 2016) comparisons can be expressed with

$$\begin{array}{cc} & H_{0}:\left\{\begin{array}{c} \lambda_{1}=\bar{\lambda }\\ \lambda_{2}=\bar{\lambda }\\ \lambda_{3}=\bar{\lambda }\\ \lambda_{4}=\bar{\lambda } \end{array}\right.\Leftrightarrow \\ & H_{0}:C\lambda =\left(\begin{array}{cccc} 1-\frac{n_{1}}{N} & -\frac{n_{2}}{N} & -\frac{n_{3}}{N} & -\frac{n_{4}}{N}\\ -\frac{n_{1}}{N} & 1-\frac{n_{2}}{N} & -\frac{n_{3}}{N} & -\frac{n_{4}}{N}\\ -\frac{n_{1}}{N} & -\frac{n_{2}}{N} & 1-\frac{n_{3}}{N} & -\frac{n_{4}}{N}\\ -\frac{n_{1}}{N} & -\frac{n_{2}}{N} & -\frac{n_{3}}{N} & 1-\frac{n_{4}}{N} \end{array}\right)\left(\begin{array}{c} \lambda_{1}\\ \lambda_{2}\\ \lambda_{3}\\ \lambda_{4} \end{array}\right)=0. \end{array}$$

Here, $\bar{\lambda }$ refers to the weighted average of the group rates $\bar{\lambda }=\sum_{j=1}^{a}\frac{n_{j}}{N}\lambda_{j}$, to which the rates $\lambda_{1},\cdots ,\lambda_{4}$ are compared. Which contrast to use depends on the research question of the experiment and cannot be recommended in a general way. A broad overview of different contrast matrices is implemented in the contrMat function in the multcomp package in R (Hothorn et al. 2008; Hothorn 2024). Next, the subsequent section will discuss multiple contrast tests and simultaneous confidence intervals for testing these general contrasts.

## 3 Multiple Contrast Tests

Testing multiple null hypotheses is a standard task in statistical practice. Often, researchers analyze several samples using an omnibus principle consisting of three consecutive steps: They first test the global null hypothesis (equality of all parameters, here the count rates $\lambda_{i}$) with an appropriate global test (usually a quadratic form, e.g., from the ANOVA family). If the global null hypothesis is rejected (e.g., at a 5% level of significance), they then perform multiple comparisons at a multiple significance level to identify the guilty levels. In the third and last step, they compute (simultaneous) confidence intervals for the effects of interest. This approach is intuitively easy but has several disadvantages: (1) the methods may neither be consonant nor coherent, that is, the global null hypothesis might get rejected, but none of the pairwise multiple comparisons and vice versa. (2) The multiple comparisons and confidence intervals might be incompatible, that is, any individual null hypothesis may be rejected, but the corresponding confidence interval contains 0—the hypothetical value of no treatment effect. Properly used closed test procedure can overcome consonance and coherency issues, however, compatible simultaneous confidence interval requires a careful investigation (Brannath and Bretz 2010). All in all, the disadvantages of the approach outweigh its advantages. To improve the procedure, we seek the origin of the omnibus procedure sketched above and find that the global test used in the first step tests can detect only one (broad) alternative, namely that at least two parameters are different. Hence, if that is not the question of interest, such a test should not be used, which also raises questions about these prominent omnibus algorithms. Multiple contrast tests overcome these limitations, and we will explain the procedures in the following: Let $C=(c_{\ell }^{\prime })$ denote a q×a contrast matrix of interest, let λ denote the vector of count rates, and $\hat{\lambda }$ the corresponding estimate. To test the individual null hypothesis $H_{0}^{\left(\ell \right)}:c_{\ell }^{\prime }\lambda =0$, consider the test statistic

(3) $$\begin{array}{c} T_{\ell }=\frac{c_{\ell }^{\prime }g\left(\hat{\lambda }\right)}{{\hat{\sigma }}_{\ell }},\;\ell =1,\text{…},q. \end{array}$$

Here, $g\left(x\right)={\left(g\left(x_{1}\right),\text{…},g\left(x_{a}\right)\right)}^{\prime }$ denotes an appropriate link function and ${\hat{\sigma }}_{\ell }^{2}=c_{\ell }^{\prime }\hat{\Sigma }c_{\ell }$ a consistent estimator of the variance of the contrast $c_{\ell }^{\prime }g\left(\hat{\lambda }\right)$. We will discuss different link functions and variance estimators below. However, even though $T_{\ell }$ follows, for large sample sizes, as N→∞, the standard normal distribution, its finite sampling distribution is unknown; therefore, appropriate approximations of its distribution in small sample sizes are needed. Furthermore, the test statistics $T_{\ell }$ and $T_{\ell^{\prime }}$ are not necessarily independent, and ignoring their dependency results in a power loss of the inferential procedure. We therefore collect the test statistics $T_{1},\text{…},T_{q}$ in the vector T that follows, for large sample sizes, as N→∞, a multivariate normal distribution with expectation 0 and correlation matrix

(4) $$\begin{array}{c} R=diag{\left(C\Sigma C^{\prime }\right)}^{-1/2}\left(C\Sigma C^{\prime }\right)diag{\left(C\Sigma C^{\prime }\right)}^{-1/2}. \end{array}$$

Suppose for a moment R is known, we then reject the individual null hypothesis $H_{0}^{\left(\ell \right)}:c_{\ell }^{\prime }\lambda =0$ at level α∈(0,1), if $|T_{\ell }|\geq z_{1-\alpha }\left(R\right)$. Here, $z_{1-\alpha }\left(R\right)$ denotes the two‐sided (1−α)100% equi‐coordinate quantile of the N(0,R) distribution. Compatible simultaneous confidence intervals for g(λ) are obtained from

$$\begin{array}{c} CI_{\ell }=\left[g\left(\hat{\lambda }\right)\pm z_{1-\alpha }\left(R\right){\hat{\sigma }}_{\ell }\right]. \end{array}$$

Finally, the global null hypothesis is rejected if any of the local null hypotheses are rejected, leading to the maximum test statistic

$$\begin{array}{c} T_{0}=\max\{|T_{1}\left|,\text{…},\right|T_{q}\left|\right\}\geq z_{1-\alpha }\left(R\right). \end{array}$$

Note that the set of test statistics and corresponding null hypotheses constitutes a joint testing family in the sense of Gabriel (1969). Since the multiple contrast test is coherent, the maximum test controls the family‐wise error rate strongly (Westfall and Young 1989). The correlation matrix R is, however, unknown in real data applications and must be either replaced by a consistent estimator or the joint distribution N(0,R) must be approximated in such a way that estimation of R is not necessary. In the following section, we will discuss several methods for the former and provide a nonparametric bootstrap algorithm for the latter.

## 4 Methods

In the following, we will discuss different estimators of the model parameters to specify the test statistics in (3). All of them are motivated by general workflows and experiences in statistical practice—they are not necessarily in line with the statistical model (1). We are interested in quantifying the impact of model misspecifications, which are often encountered in practice, on the Type I error rates of the tests and their powers to detect selected alternatives.

### 4.1 Linear Models

#### 4.1.1 Homoscedastic Variances

Many modern procedures exist for analyzing and modeling count data, particularly generalized linear models for various distributions. All of these, however, are regression models and usually are not the first method of choice when the standard ANOVA task comes into question, namely comparing several means of independent groups. In statistical practice, dispersion is usually modeled as global effect and hence, assuming a standard ANOVA model $X_{ik}=\lambda_{i}+\varepsilon_{ik}$, where $\varepsilon_{ik}∼N\left(0,\sigma^{2}\right)$ and estimating $\lambda_{i}$ with the empirical means (as above) and $\sigma^{2}$ with the pooled empirical variance

$${\hat{\sigma }}_{pool}^{2}=\frac{1}{N-a}\sum_{i=1}^{a}\left(n_{i}-1\right){\hat{\sigma }}_{i}^{2}$$

sounds intuitive, and the corresponding test statistics are given by

$$\begin{array}{cc} T_{0}^{\mathrm{hom}} & =\max\{|T_{1}^{\mathrm{hom}}|,\cdots ,|T_{q}^{\mathrm{hom}}|\},\;\text{where}\;\\ T_{\ell }^{\mathrm{hom}} & =\frac{c_{\ell }^{\prime }\hat{\lambda }}{{\hat{\sigma }}_{\mathrm{pool}}\sqrt{c_{\ell }^{\prime }\mathrm{diag}\left(\frac{1}{n_{1}},\cdots ,\frac{1}{n_{a}}\right)c_{\ell }}},\;\ell =1,\cdots ,q. \end{array}$$

In this case, the correlation matrix R in (4) is known and only depends on the chosen contrast matrix C and sample sizes $n_{1},\text{…},n_{a}$.

#### 4.1.2 Heteroscedastic Variances

Modeling count data by a linear model with homoscedastic variances creates doubts. Therefore, we investigate the behavior of multiple contrast tests, allowing for heteroscedasticity

(5) $$\begin{array}{cc} T_{0}^{\mathrm{het}} & =\max\left\{|T_{1}^{\mathrm{het}}\left|,\cdots ,\right|T_{q}^{\mathrm{het}}|\right\},\;\text{where}\\ T_{\ell }^{\mathrm{het}} & =\frac{c_{\ell }^{\prime }\hat{\lambda }}{\sqrt{c_{\ell }^{\prime }{\hat{\Sigma }}_{\mathrm{HC}}c_{\ell }}},\;\ell =1,\cdots ,q, \end{array}$$

where ${\hat{\Sigma }}_{HC}$ denotes White's heteroscedasticity‐consistent sandwich‐type estimator (White 1980; Zeileis 2006; Herberich et al. 2010). Under heteroscedasticity, the correlation matrix R in (4) is unknown and depends on the chosen contrast matrix C, unknown variances $\sigma_{1}^{2},\text{…},\sigma_{a}^{2}$, and sample sizes $n_{1},\text{…},n_{a}$, and thus must be replaced by a consistent estimator, for example,

$$\begin{array}{c} \hat{R}=diag{\left(C{\hat{\Sigma }}_{HC}C^{\prime }\right)}^{-1/2}\left(C{\hat{\Sigma }}_{HC}C^{\prime }\right)diag{\left(C{\hat{\Sigma }}_{HC}C^{\prime }\right)}^{-1/2}. \end{array}$$

Note that White's estimator is not only consistent group‐wise but also under complete heteroscedasticity in the sense that the variance of every random variable $X_{ik}$ may be subject‐specific, that is, $Var\left(X_{ik}\right)=\sigma_{ik}^{2}$. Estimating the variances group‐wise leads to similar conclusions, but we omit these results. We estimated model parameters of linear models with the function glht() from the R package multcomp (Hothorn 2024).

#### 4.1.3 Data Transformations

Count data often follow a skewed distribution. Therefore, symmetrizing data transformations are frequently used in statistical practice to make the use of t‐test‐type procedures more plausible, even though the approach is critically discussed (O'Hara and Kotze 2010; Ives 2015; Warton 2018). Well‐known are the f(x)=log(x) and square‐root transformations $f\left(x\right)=\sqrt{x}$, typically encountered in situations with and without a large number of zeros, respectively. To remove zeros and make the log‐transformation applicable, a pragmatic compromise between these two is to add a small constant (e.g., 1) to each count and transform those afterward. We will investigate the impact of two different data transformations, namely

(6) $$\begin{array}{ccc} Y_{ik} & = & log(X_{ik}+1)\;\text{and} \end{array}$$

(7) $$\begin{array}{ccc} Y_{ik} & = & \sqrt{X_{ik}+0.5}, \end{array}$$

on the behavior of the multiple contrast tests under heteroscedasticity as given in (5) in extensive simulation studies in Section 5. Siegfried and Hothorn (2020) proposed a framework for transforming count data, where the kind of transformation is directly estimated from the data without an a priori definition. However, since we faced numerical instabilities in our extensive simulation study focusing on small sample sizes, we refrain from data‐driven transformations in these situations.

### 4.2 Generalized Linear Models

Strictly speaking, evaluating count data using classical multiple contrast tests, as discussed above, is only valid under the assumption of normality or with large sample sizes. Generalized linear models employ a distinct modeling approach, utilizing the characteristics of an assumed probability distribution for the counts. It has been shown previously that multiple comparisons can be used in generalized linear models (Hothorn et al. 2008). In the specific situation considered here, the models can be summarized by estimating

$$\begin{array}{c} E\left(X_{ik}|\text{group membership}\;\right)=\log\left(\lambda_{i}\right),\;\;i=1,\text{…},a, \end{array}$$

using distributional quantities within the maximum‐likelihood estimation of the model parameters $\lambda_{1},\text{…},\lambda_{a}$ and their variances, along with further quantities, for example, overdispersion in Negative Binomial models. The multiple contrast tests are given by

(8) $$\begin{array}{cc} T_{0}^{\mathrm{GLM}} & =\max\left\{|T_{1}^{\mathrm{GLM}}\left|,\cdots ,\right|T_{q}^{\mathrm{GLM}}|\right\},\;\text{where}\\ T_{\ell }^{\mathrm{GLM}} & =\frac{c_{\ell }^{\prime }g\left(\hat{\lambda }\right)}{\sqrt{c_{\ell }^{\prime }{\hat{\Sigma }}_{\mathrm{GLM}}c_{\ell }}},\;\ell =1,\cdots ,q. \end{array}$$

Here, $g\left(\hat{\lambda }\right)={\left(\log\left({\hat{\lambda }}_{1}\right),\text{…},\log\left({\hat{\lambda }}_{a}\right)\right)}^{\prime }$ denotes the log‐transformed estimators and ${\hat{\Sigma }}_{GLM}$ denotes the estimated covariance matrix of $g\left(\hat{\lambda }\right)$ obtained from the GLM. We investigate the impact of differently assumed distributions (Poisson, Negative Binomial, and Quasi‐Poisson) on the behavior of $T_{0}^{GLM}$ in (8) in extensive simulation studies (see Section 5), respectively. Throughout, we estimated the model parameters for generalized linear models using the R package emmeans (Lenth 2024).

### 4.3 A Nonparametric Bootstrap Test

Although each of the introduced procedures is based on a specific statistical model (linear vs. generalized linear), they appear to be similar yet still employ substantially different SEs. In small sample sizes, verifying distributional assumptions as a prerequisite for GLM is nearly impossible, making their applicability questionable in these situations. Indeed, if the model is misspecified, the variance estimators are biased, resulting in inaccurate Type I errors of the test procedures. Therefore, we introduce a similar procedure that is valid in the general semiparametric model (1) without postulating specific distributional assumptions or linear relationships in the data (and their parameters). To test the null hypotheses $H_{0}:C\lambda =0$, consider the maximum statistic

$$\begin{array}{cc} T_{0} & =\max\left\{|T_{1}\left|,\cdots ,\right|T_{q}|\right\},\;\text{where}\;\;\\ T_{\ell } & =\frac{c_{\ell }^{\prime }g\left(\hat{\lambda }\right)}{\sqrt{c_{\ell }^{\prime }\mathrm{diag}\left(\frac{{\hat{\sigma }}_{1}^{2}}{n_{1}{\hat{\lambda }}_{1}^{2}},\cdots ,\frac{{\hat{\sigma }}_{a}^{2}}{n_{a}{\hat{\lambda }}_{a}^{2}}\right)c_{\ell }}},\;\ell =1,\cdots ,q, \end{array}$$

denote GLM‐type test statistics with the variances being estimated by their empirical counterparts. Its distribution follows, for large sample sizes, as N→∞ under **A1** and **A2**, a multivariate normal distribution with expectation 0 and correlation matrix R being as given in (4). In comparison with the GLM‐based statistics discussed above in (8), we estimate the variances of the effect estimators in a general way. However, extensive simulation studies indicate that a liberal behavior occurs in the case of small sample sizes: It over‐rejects the null hypothesis. We strive for a better approximation of its joint distribution in these cases and equip the method with a nonparametric bootstrap approach: Draw $n_{i}$ observations $X_{ik}^{*}$ from each sample $X_{i1},\text{…},X_{in_{i}},i=1,\text{…},a$, with replacement separately and compute the maximum statistic

$$\begin{array}{cc} T_{0}^{*} & =\max\left\{|T_{1}^{*}\left|,\cdots ,\right|T_{q}^{*}|\right\},\;\text{where}\;\;\\ T_{\ell }^{*} & =\frac{c_{\ell }^{\prime }\left(g\left({\hat{\lambda }}^{*}\right)-g\left(\hat{\lambda }\right)\right)}{\sqrt{c_{\ell }^{\prime }\mathrm{diag}\left(\frac{{\hat{\sigma }}_{1}^{{*}^{2}}}{n_{1}{\hat{\lambda }}_{1}^{{*}^{2}}},\cdots ,\frac{{\hat{\sigma }}_{a}^{{*}^{2}}}{n_{a}{\hat{\lambda }}_{a}^{{*}^{2}}}\right)c_{\ell }}},\;\ell =1,\cdots ,q. \end{array}$$

Here, ${\hat{\lambda }}^{*}={\left({\hat{\lambda }}_{1}^{*},\text{…},{\hat{\lambda }}_{a}^{*}\right)}^{\prime }$ denotes the vector of estimators ${\hat{\lambda }}_{i}^{*}=n_{i}^{-1}\sum_{k=1}^{n_{i}}X_{ik}^{*}$ and ${\hat{\sigma }}_{i}^{*,2}={\left(n_{i}-1\right)}^{-1}\sum_{k=1}^{n_{i}}{\left(X_{\mathrm{ik}}^{*}-{\hat{\lambda }}_{i}^{*}\right)}^{2}$, the empirical variance of the resampling variables $X_{i1}^{*},\text{…},X_{in_{i}}^{*}$, respectively. As a link function $g\left(\right)$, we also used the log. The critical value $z_{1-\alpha }\left(R\right)$ (equi‐coordinate quantile from the joint distribution of the vector of test statistics) or the multiplicity‐adjusted *p*‐values for each hypothesis can now be estimated from the resampling distribution of $T_{0}^{*}$. To do so, repeat the above steps a large number of times, for example, $n_{boot}=5K$ times, save the values of $T_{0}^{*}$ in $T_{0}^{\left(1,*\right)},\text{…},T_{0}^{\left(n_{boot},*\right)}$, say, and estimate the quantile by their empirical (1−α)·100% quantile, respectively. Equivalently, we can estimate the adjusted *p*‐values accordingly; for instance, for the global null hypothesis by

$$\begin{array}{c} {\text{p-value}}_{0}=\frac{1}{n_{boot}}\sum_{j=1}^{n_{boot}}I\left(T_{0}^{\left(j,*\right)}\geq T_{0}\right). \end{array}$$

We note that the resampling distribution mimics the limiting distribution of $T_{0}$ for large sample sizes, which follows the same arguments as used in the proof of Theorem 1 in Pauly et al. (2015). For similar results, see Munko et al. (2024). In the next section, we will elaborate on its qualities (Type I error rate control in various scenarios and its power to detect selected alternatives) in small sample sizes in extensive simulation studies.

**Remark:** Note that the asymptotic distribution of the test statistic $T_{0}$ is non‐pivotal and depends on the unknown correlation matrix R. Therefore, using permutations, or drawing with replacement from the joint sample, instead of group‐wise drawing with replacement, will not directly lead to a valid resampling test in the general scenario considered here, because the correlation matrix of the resampled test statistics differs from R, even asymptotically. For similar arguments, we refer to Pauly et al. (2015).

Additionally, note that the bootstrap test is constructed in a way that ensures subset pivotality is satisfied (Westfall and Young 1993; Davison and Hinkley 1997). We draw with replacement from each group separately to ensure that the limiting joint limit distributions of the test statistics T and $T^{*}$ coincide. The asymptotic equivalence holds for any subset of the test statistics. In particular, the distribution of the tests is derived under arbitrary but fixed alternatives; we do not assume that any null hypothesis is true at any stage. Therefore, for every possible subset of true null hypotheses, the joint distribution of the corresponding test statistics under the intersection null hypothesis is identical to their distribution under the complete null hypothesis (where all hypotheses are true), and therefore, subset pivotality holds. If we resampled differently, for example, drawing with replacement from the joint sample, subset pivotality would in general not hold due to potentially different limiting correlation matrices for different hypotheses.

## 5 Simulation Study

All the methods investigated above are asymptotic and thus accurately control the Type I error level α∈(0,1) when the sample sizes are large. The same can be said about their powers to detect selected alternatives; they will detect them with high probability (one or close to one) in these situations. Of significant interest is investigating their properties when sample sizes are small. Since the exact distributions of the tests are unknown in finite sample size settings, we conducted extensive simulation studies to assess their Type I error rate control and power to detect selected alternatives when sample sizes are as small as $n_{i}∼6$. Since the wording “count data distribution” is rather generic and comprises a manifold of different possible models, we will examine the impact of varying count data distributions on the tests' properties in the simulation studies (Johnson et al. 2005). We discuss the main properties of the selected distributions in the following subsection.

### 5.1 Distributions for Count Data

To cover various distributional shapes and dispersions, we generated data from three different distributions: the Poisson, Negative Binomial, and Conway–Maxwell–Poisson (CMP) distributions. Note that the Negative Binomial distribution can model only overdispersed count data; the CMP can model both over‐ and underdispersed count data. The Poisson and Negative Binomial distributions are frequently used and well‐known in statistical practice. Note that generalized linear models in these families are implemented in almost every statistical software package. The CMP is less prominent but equally important. Their key properties, along with R commands, are given below:

#### 5.1.1 Poisson (POI)

If X∼Pois(λ), then λ=E(X)=V(X) (equidispersion assumption), and hence, the dispersion cannot be modeled. In R, data from a Poisson distribution (POI) can be generated with the function rpois().

#### 5.1.2 Negative Binomial (NB)

The Negative Binomial distribution (NB) relaxes the equidispersion assumption to E(X)≤V(X); hence, overdispersed data can be modeled. We generated Negative Binomial data in R with the mean parameterization option of the rnbinom() function. Here, the expectation μ and dispersion are defined by mu and size. The corresponding variance is given by $V\left(X\right)=\mu +\frac{\mu^{2}}{size}$.

#### 5.1.3 Conway–Maxwell–Poisson (CMP)

The Conway–Maxwell–Poisson distribution (CMP) can model over‐ and underdispersion (Shmueli et al. 2005). It is a generalization of the Poisson, Bernoulli, and geometric distributions. Alwan (2023) presented a mean parameterization, implemented in the R package mpcmp (Fung et al. 2022), which was used for implementation in this simulation study. The function rcomp() also needs two parameters: the expected value E(X)=μ and the dispersion parameter ν. With ν<1, overdispersed data can be simulated, and ν>1 results in underdispersed data. Next, we discuss the parameter settings of the simulation study.

### 5.2 Settings

One of the main interests in the simulation study is investigating the impact of data distributions on the properties of the tests in small samples, particularly the tests' behavior in positive and negative sample sizes and variance (dispersion) allocations, also known as positive and negative pairings in unbalanced designs. We generated data from the three distributions POI, NB, and CMP with three different rates $\lambda_{i}\in \left\{1,6,10\right\}$ and varying dispersion parameters (details are provided below). Besides these, other factors, such as the number of groups and the contrast (matrix), are also interesting. These lead to countless designs and parameter constellations. We, therefore, select many different designs comprising balanced and unbalanced designs involving a∈{3,4} groups and three different contrasts (matrices) C∈{Dunnett, Tukey, Grand-Mean}. For ease of reading, we will display simulation results for settings involving four groups in the main document; other results will be presented in the Supporting Information. Table 1 shows an overview of the selected settings.

**TABLE 1:** Overview of simulated designs with sample sizes $n_{1}={\left(6,6,6,6\right)}^{\prime },n_{2}={\left(6,10,10,16\right)}^{\prime }$, and m∈{0,2,4,⋯,24}.

Setting	Distrib.	Sample sizes	Dispersion	Dispersion parameter	Interpretation
1	POI	$n=n_{1}+m$	Equidispersed	—	Balanced homosc.
2	POI	$n=n_{2}+m$	Equidispersed	—	Unbalanced homosc.
3	CMP	$n=n_{1}+m$	Overdispersed	$\nu ={\left(0.5,0.5,0.5,0.5\right)}^{\prime }$	Balanced homosc.
4	CMP	$n=n_{1}+m$	Underdispersed	$\nu ={\left(2,2,2,2\right)}^{\prime }$	Balanced homosc.
5	CMP	$n=n_{1}+m$	Overdispersed	$\nu ={\left(0.2,0.2,0.5,0.5\right)}^{\prime }$	Balanced heterosc.
6	CMP	$n=n_{1}+m$	Over‐ & underd.	$\nu ={\left(0.2,0.5,2,2\right)}^{\prime }$	Balanced heterosc.
7	CMP	$n=n_{2}+m$	Overdispersed	$\nu ={\left(0.5,0.5,0.2,0.2\right)}^{\prime }$	Positive pairing
8	CMP	$n=n_{2}+m$	Over‐ & underd.	$\nu ={\left(2,2,0.5,0.2\right)}^{\prime }$	Positive pairing
9	CMP	$n=n_{2}+m$	Overdispersed	$\nu ={\left(0.2,0.2,0.5,0.5\right)}^{\prime }$	Negative pairing
10	CMP	$n=n_{2}+m$	Over‐ & underd.	$\nu ={\left(0.2,0.5,2,2\right)}^{\prime }$	Negative pairing
11	NB	$n=n_{1}+m$	Overdispersed	$\mathrm{size}={\left(3,3,3,3\right)}^{\prime }$	Balanced homosc.
12	NB	$n=n_{2}+m$	Overdispersed	$\mathrm{size}={\left(5,3,3,2\right)}^{\prime }$	Positive pairing
13	NB	$n=n_{2}+m$	Overdispersed	$\mathrm{size}={\left(0.75,2,2,3\right)}^{\prime }$	Negative pairing
14	NB	$n=n_{2}+m$	Overdispersed	$\mathrm{size}={\left(2,3,3,5\right)}^{\prime }$	Negative pairing

*Note:* Rate parameter $\lambda_{i}\in \left\{1,6,10\right\}$ with i∈{1,2,3,4}, $n_{j}+m$ means that each component of $n_{j}$ increases by m.

In balanced designs, we investigate samples sizes $n_{1}={\left(n_{1},n_{2},n_{3},n_{4}\right)}^{\prime }={\left(6,6,6,6\right)}^{\prime }$ and increase each component by a constant m∈{0,2,4,⋯,24}, similarly for unbalanced settings with $n_{2}={\left(n_{1},n_{2},n_{3},n_{4}\right)}^{\prime }={\left(6,10,10,16\right)}^{\prime }$ to illustrate the tests' behavior in increasing sample sizes. The different combinations of dispersion parameters within a setting are given in Table 1, including overdispersion and combinations of over‐ and underdispersion. Table A1 provides an overview of the simulated distributions used within the settings, characterized by the following parameters: variance, proportion of zeros, and the variance‐to‐mean ratio, which serves as an estimate of overdispersion. Each simulation setting was investigated using $n_{sim}=10K$ simulation runs and $n_{boot}=5K$ bootstrap runs. The simulated Type I error rates refer to the nominal significance level of α=5%. We discuss the results and our main findings in the subsequent section.

**TABLE 2:** Summary of the Type I error rate control regarding the simulated settings.

Characteristic: Sensitive to	boot	hom	het	het_log	het_sqrt	poi	nb	q_poi
# groups	No	No	No	No	No	No	No	No
Type of contrast	No	Yes	No	Yes	Yes	Yes	Yes	Yes
Data distribution	No	Yes	No	Yes	Yes	Yes	Yes	Yes
Overdispersion	No	Yes	No	Yes	Yes	Yes	No	No
Underdispersion	No	Yes	No	Yes	Yes	Yes	Yes	No
Small sample sizes combined with small lambda	Yes	Yes	Yes	Yes	Yes	Yes	Yes	Yes
Small sample sizes combined with higher lambda	Yes	Yes	Yes	Yes	Yes	Yes	Yes	Yes

**TABLE 3:** Characteristics of the distribution of the number of visits stratified by age.

Age group	Sample size	Estimated rate ${\hat{\lambda }}_{i}$	Sample variance	Proportion of zeros	$\frac{\mathrm{Variance}}{\mathrm{Mean}}$
20–29	10	6.8	21.1	0.10	3.1
30–39	28	6.3	47.0	0.14	7.4
40–49	39	5.7	52.5	0.18	9.1
50–60	35	6.2	32.7	0.09	5.3

**TABLE 4:** Results for application of the different methods using Dunnett as contrast.

Method	Scale	Contrast	Estimate	SE	Statistic	*p*‐Value	95% CI
boot	log(λ)	2–1	−0.07	0.30	0.25	0.99	(−0.83; 0.69)
boot		3–1	−0.17	0.29	0.57	0.89	(−0.92; 0.59)
boot		4–1	−0.10	0.26	0.37	0.97	(−0.78; 0.58)
hom	original	2−1	−0.48	2.40	0.20	0.99	(−5.99; 5.03)
hom		3−1	−1.06	2.30	0.46	0.90	(−6.36; 4.24)
hom		4−1	−0.63	2.33	0.27	0.98	(−5.99; 4.73)
het	original	2−1	−0.48	2.02	0.24	0.99	(−5.23; 4.27)
het		3−1	−1.06	1.93	0.55	0.88	(−5.59; 3.48)
het		4−1	−0.63	1.82	0.35	0.97	(−4.90; 3.64)
het_log	log(x+1)	2−1	−0.21	0.32	0.64	0.80	(−0.95; 0.54)
het_log		3−1	−0.35	0.31	1.13	0.46	(−1.08; 0.37)
het_log		4−1	−0.18	0.31	0.59	0.83	(−0.90; 0.54)
het_sqrt	sqrt(x+0.5)	2−1	−0.20	0.39	0.51	0.89	(−1.11; 0.71)
het_sqrt		3−1	−0.35	0.38	0.94	0.60	(−1.23; 0.52)
het_sqrt		4−1	−0.19	0.37	0.52	0.88	(−1.05; 0.67)
poi	log(λ)	2−1	−0.07	0.14	0.51	0.88	(−0.40; 0.25)
poi		3−1	−0.17	0.14	1.22	0.40	(−0.48; 0.15)
poi		4−1	−0.10	0.14	0.70	0.76	(−0.41; 0.22)
nb	log(λ)	2−1	−0.07	0.37	0.20	0.99	(−0.92; 0.77)
nb		3−1	−0.17	0.36	0.47	0.90	(−0.99; 0.65)
nb		4−1	−0.10	0.36	0.27	0.98	(−0.92; 0.73)
q_poi	log(λ)	2−1	−0.07	0.38	0.19	0.99	(−0.93; 0.79)
q_poi		3−1	−0.17	0.37	0.46	0.90	(−1.00; 0.66)
q_poi		4−1	−0.10	0.37	0.26	0.98	(−0.94; 0.74)

### 5.3 Results

**FIGURE 1:**
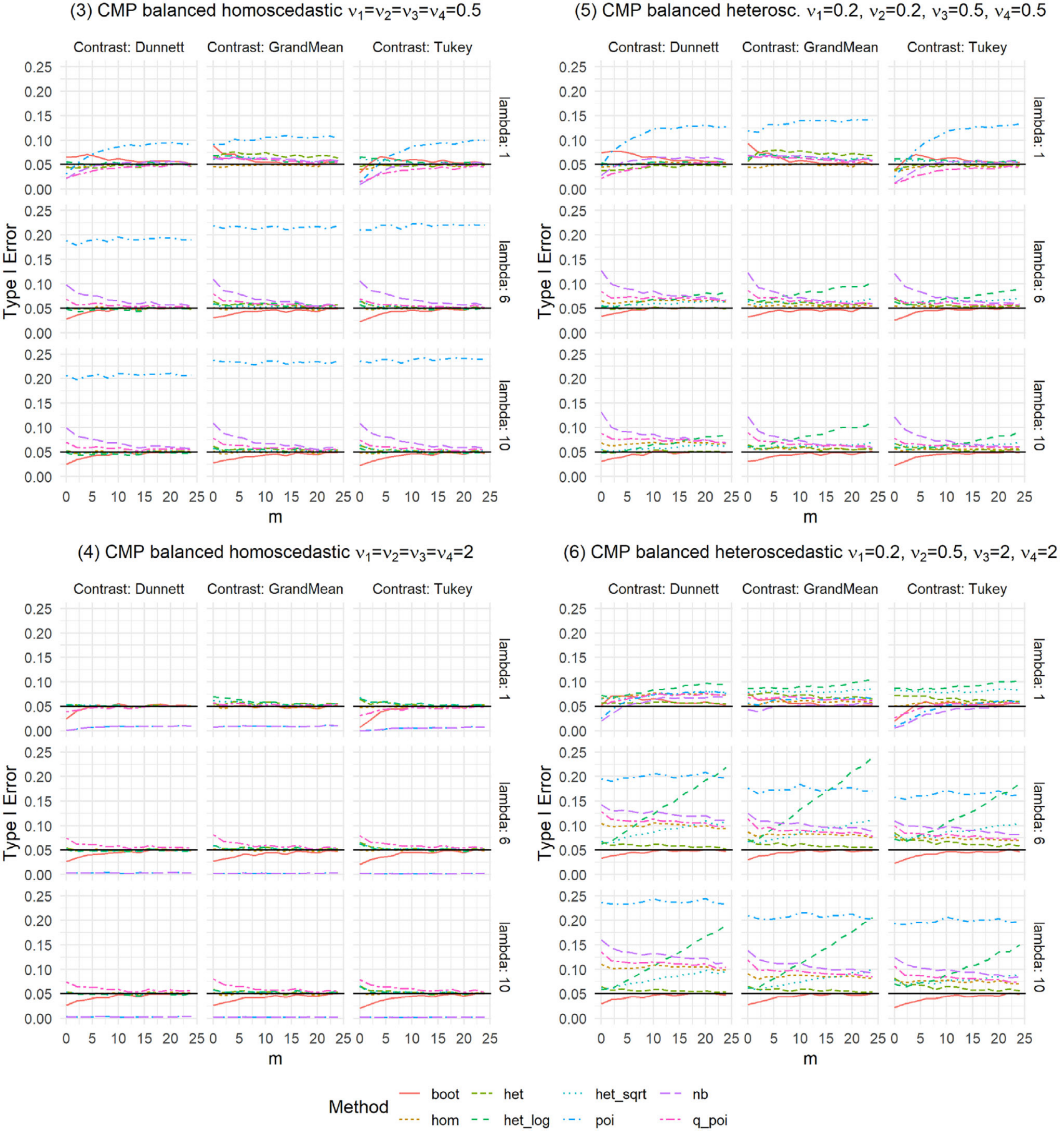
Type I error simulation for balanced settings of Conway–Maxwell Poisson distributed data with overdispersion (ν∈{0.2,0.5}), underdispersion (ν=2), and combinations of both.

**FIGURE 2:**
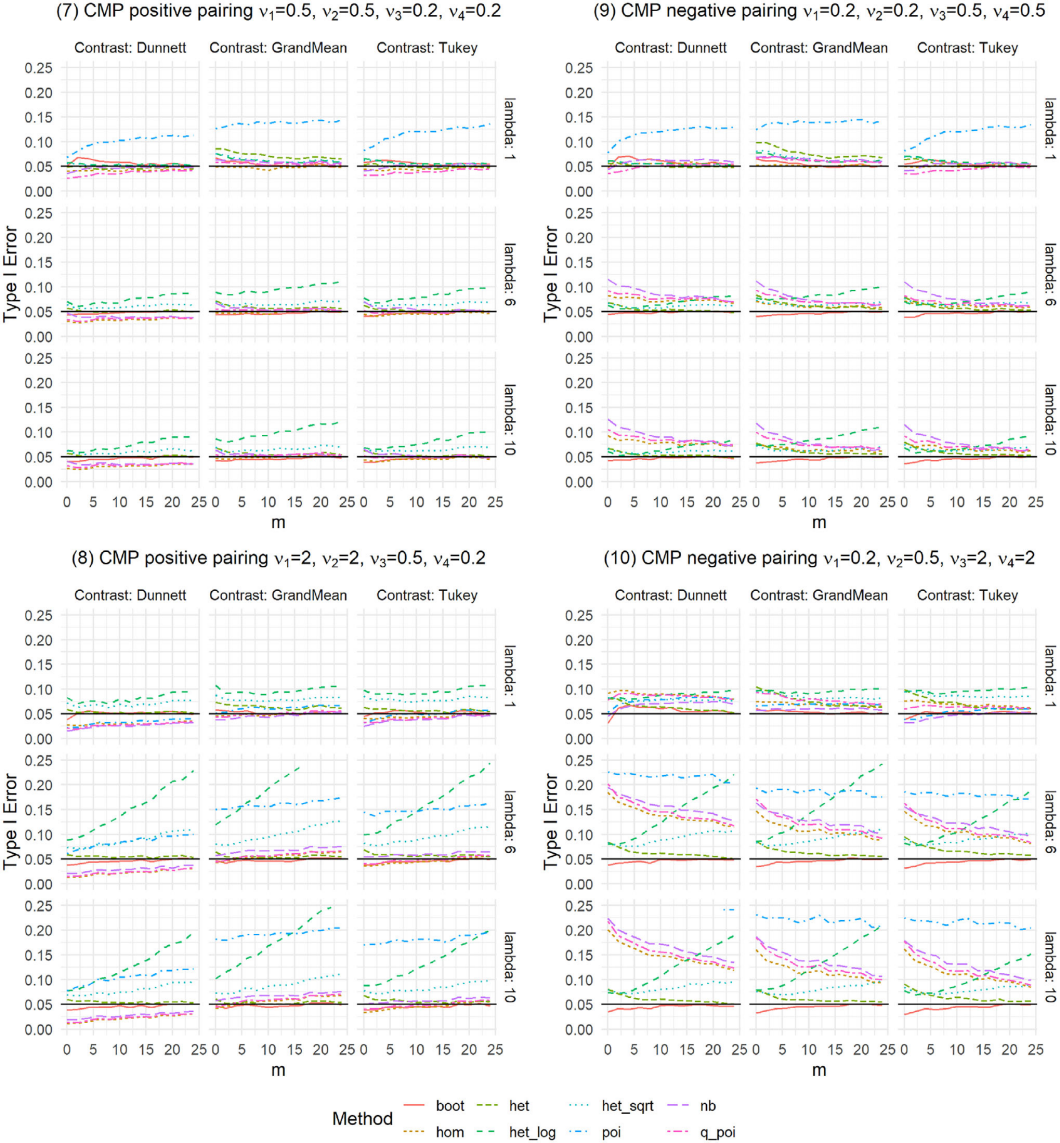
Type I error simulation for unbalanced settings of Conway‐Maxwell Poisson distributed data with overdispersion (ν∈{0.2,0.5}), underdispersion (ν=2), and combinations of both.

**FIGURE A1:**
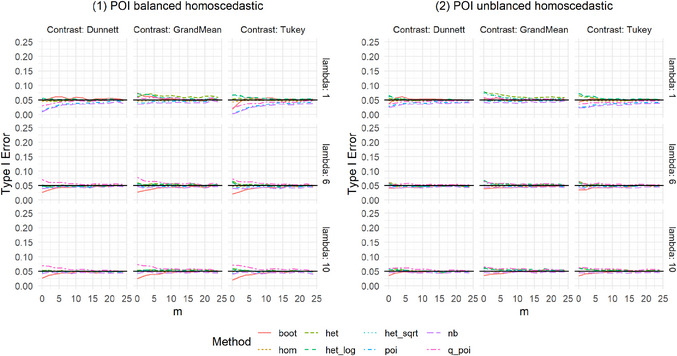
Type I error simulation for Poisson distributed data.

**FIGURE A2:**
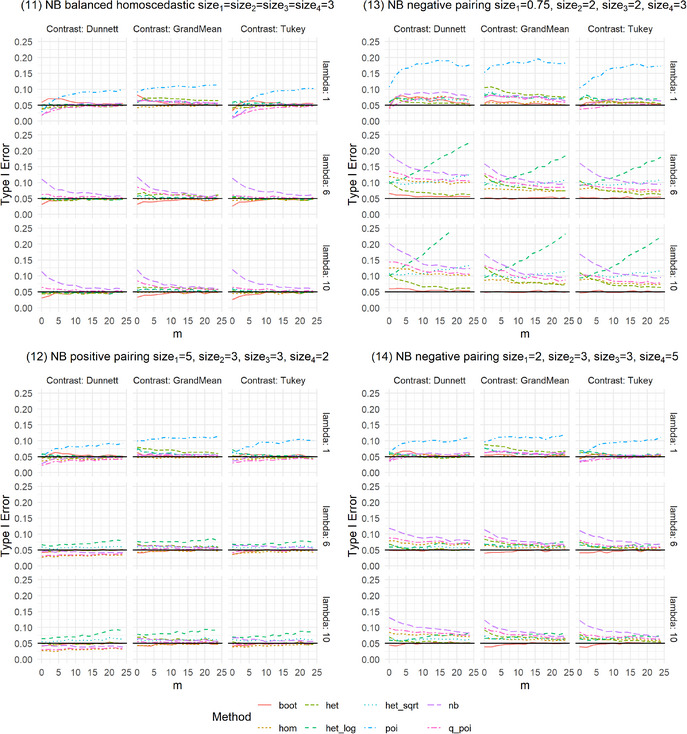
Type I error simulation for Negative Binomial distributed data.

We present the results of the settings introduced in Table 1, which are shown in Figures 1 and 2 and A1 and A2. The Type I error was examined for the eight methods introduced in Section 4: a nonparametric bootstrap test (boot), linear models with homoscedastic (hom) and heteroscedastic (het) variances as well as with heteroscedastic variances after data transformation using a log transformation as in formula 6 (het_log) or a square root transformation as in formula 7 (het_sqrt) and generalized linear models assuming the distributions Poisson (poi), Negative Binomial (nb), or Quasi‐Poisson (q_poi). An overview of the results is shown in Table 2.

**FIGURE 3:**
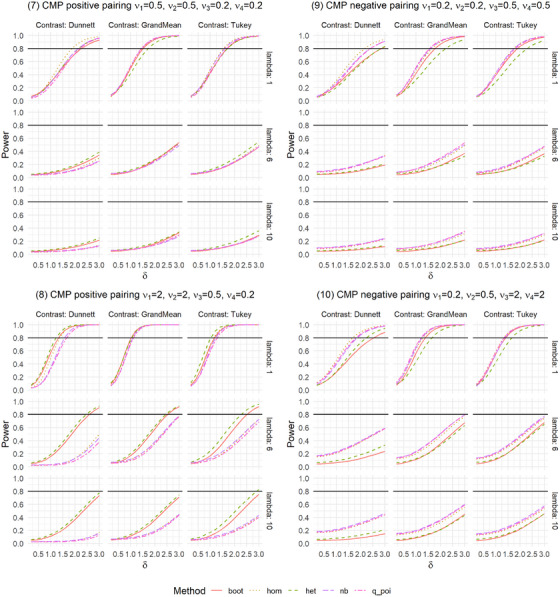
Power simulation for Conway–Maxwell Poisson distributed data with positive and negative pairing.

**FIGURE 4:**
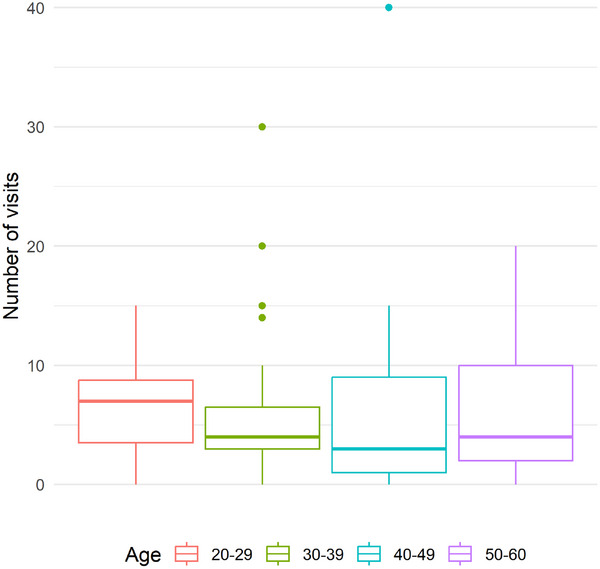
Boxplots for number of visits stratified by age.

In general, no single method controls the Type I error best in all examined settings, but het and boot perform pretty well in all settings. In settings with overdispersion, poi gets extremely liberal with up to 81% Type I error rates (Type I error is only displayed up to 25%). In most scenarios considered here, the type of contrast does not seem to impact the Type I error rate control of the methods.

For homoscedastic Setting 1 (balanced) and Setting 2 (unbalanced) with Poisson distributed data, all eight methods control the Type I error well (Figure A1). Regarding balanced homoscedastic settings with overdispersion, as in Settings 3 (CMP distributed) and 11 (NB distributed), one can see that poi becomes very liberal, especially with higher lambda values, leading to higher overdispersion (Figures A2). In addition, boot is somewhat liberal for small lambda = 1 combined with small sample sizes, and nb, and q_poi are slightly liberal for higher lambda and small sample sizes. Here, nb is more liberal than q_poi with a Type I error of around 10%. Otherwise, poi and nb are very conservative in balanced homoscedastic Setting 4 with underdispersion (CMP distributed), with Type I error near zero. In this case, q_poi is again slightly liberal, and boot is slightly conservative for very small sample sizes. All other methods effectively control the Type I error in the described balanced homoscedastic settings.

More methods result in higher Type I error rates in settings with heteroscedastic variances. Starting with balanced heteroscedastic CMP‐distributed settings, one can see that poi is constantly highly liberal and het_log and het_sqrt get more liberal the higher the sample sizes, het_log results in higher increases in Type I error than het_sqrt (Figure 1, Settings 5 and 6). Moreover, nb, q_poi, and hom are liberal but get less liberal with higher sample sizes. Also, het shows a slightly liberal behavior for all‐pairwise contrasts (Tukey‐type) in Setting 6, where over‐ and underdispersed variances are combined. For lambda = 1, boot is again slightly liberal for small sample sizes and somewhat conservative in the case of higher lambdas and very small sample sizes. The results for heteroscedastic variances are more extreme when combining over‐ and underdispersed distributions, as in Setting 6, compared to Setting 5, which only includes overdispersed distributions. Combining heteroscedastic variances with different group sizes results in positive and negative pairings: Settings 7 and 8 show positive pairing for CMP‐distributed data (Figure 2). The findings for het_log and het_sqrt are similar to those for balanced heteroscedastic settings; these methods result in liberal Type I errors. The results for poi are again almost always highly liberal. In contrast to the balanced settings, the behavior of nb and q_poi ranges for positive pairing from conservative to slightly liberal. In Setting 8, differences between the contrasts can be recognized: nb, q_poi, and hom are more conservative for Dunnett than for Tukey and Grand‐Mean. Setting 12 shows results for positive pairing for NB‐distributed data (Figure A2), with less extreme deviations. Settings 9 and 10 for CMP‐distributed data and Settings 13 and 14 for NB‐distributed data give results for negative pairing. For CMP‐distributed data, poi is again highly liberal. In addition, nb, q_poi, and hom are pretty liberal, especially for smaller sample sizes and in settings combining over‐ and underdispersed data. The findings for het_log and het_sqrt are the same as for balanced heteroscedastic settings. Here, boot and het adhere to the Type I error best. Het is slightly liberal in small sample sizes, while boot is somewhat liberal in settings with small sample sizes and small lambda. For NB‐distributed data, the behavior of the methods is similar to CMP‐distributed data. The allocation of dispersion and rate parameters strongly impacts the Type I error rate control for most linear models (hom, het_log, het_sqrt) and generalized linear models (poi, nb, q_poi). This is especially visible in settings with negative pairing. The Supporting Information shows results for three groups with similar settings, utilizing identical distributions but with one group less. The results for three groups are similar to those for four groups.

### 5.4 Power

**FIGURE A3:**
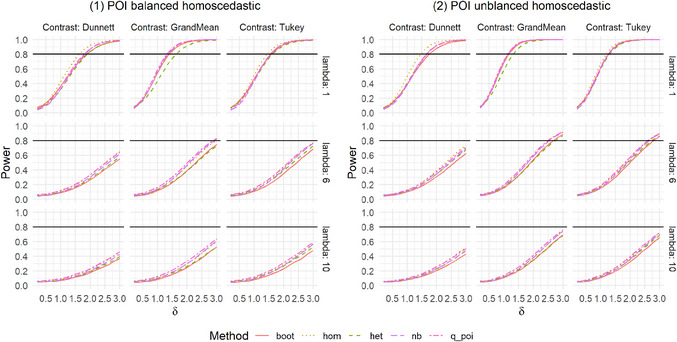
Power simulation for Poisson distributed data.

**FIGURE A4:**
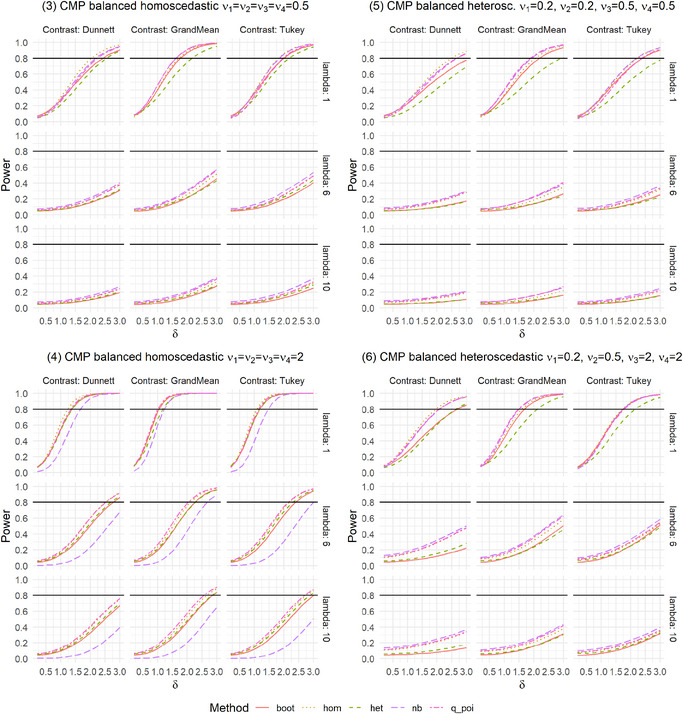
Power simulation for Conway–Maxwell Poisson distributed data with balanced settings.

**FIGURE A5:**
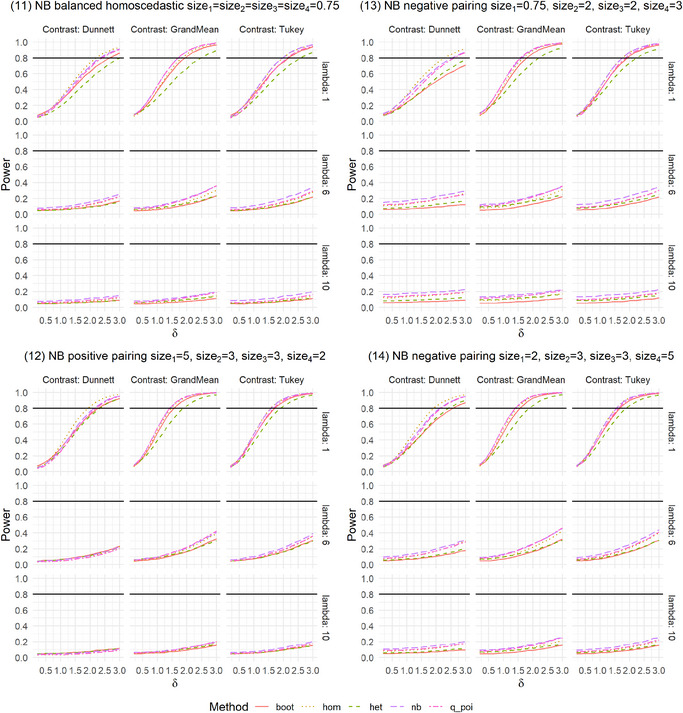
Power simulation for Negative Binomial distributed data.

In practice, not only is the Type I error rate of interest, but the power of a test is also essential. Therefore, we conducted a simulation study regarding the global power for the settings described in Table 1. We investigated these settings for selected sample sizes of $n={\left(12,12,12,12\right)}^{\prime }$ for balanced settings and $n={\left(12,16,16,22\right)}^{\prime }$ for unbalanced settings, which corresponds to m=6. Since poi, het_log, and het_sqrt are generally not recommended due to highly inflated Type I error rates in many settings, we omitted these methods from the power simulations. As a selected alternative, we investigated a difference in the second group with ${\tilde{\lambda }}_{2}=\lambda_{2}+\delta$ and δ∈{0.2,0.4,…,3}. As we are testing multiple hypotheses, additional power aspects are of interest to get a comprehensive impression of the method's behavior. For this purpose, we also examined any‐pairs and all‐pairs power. Any‐pairs power is the probability to reject at least one false null hypothesis, and the all‐pairs power is the probability to reject all false null hypotheses. Therefore, we investigated scenarios with two and three groups having different rate parameter, namely (1) ${\tilde{\lambda }}_{1}={\tilde{\lambda }}_{2}=\lambda ,{\tilde{\lambda }}_{3}=\lambda +1.5,{\tilde{\lambda }}_{4}=\lambda +2.5$ and (2) ${\tilde{\lambda }}_{1}=\lambda ,{\tilde{\lambda }}_{2}=\lambda +1.5,{\tilde{\lambda }}_{3}=\lambda +2.5,{\tilde{\lambda }}_{4}=\lambda +3$. We explored these powers for λ=6 and the contrasts Dunnett and Tukey. The results are given in Figures 3, A3, A4, A5, and Supporting Information tables.

The global power is generally similar for the five methods in most settings. Only in some settings are differences between the contrasts recognizable. In settings where nb and q_poi were very conservative, for example, in Setting 8 with positive pairing (Figure 3), the power is also smaller. This difference between the methods is higher for the Dunnett type than for the other two contrasts. In Settings 9 and 10 with negative pairing, boot and het have slightly smaller power, but were also the two methods that controlled the Type I error rate best. As expected, liberal methods result in higher power.

Supporting Information Tables S1– S4 show the results for any‐pairs and all‐pairs power. In situation (1) with one remaining true null hypothesis, any‐pairs power behaves analogously to the global power. In situation (2), where all null hypotheses are false, any‐pairs power equals the global power. All‐pairs power is relatively small for all investigated settings, given the small sample sizes. For Tukey‐type contrasts, all‐pairs power is nearly zero for these settings. Again, some differences between the methods are recognizable, which are analogous to the differences seen for the global power and the Type I error.

## 6 Real Data Application

The data set “badhealth” for this application is from a German health survey and included in the R package COUNT (Hilbe 2016). The outcome is the count variable numvisit, the number of visits to the doctor during the year 1998. We examine the group of patients that claim to be in bad health, n=112. We are interested in differences in the number of visits between four age groups defined post hoc; the data set includes age as a continuous variable. Figure 4 shows boxplots for the number of visits in each age group, and Table 3 gives information on sample sizes, the estimated rate ${\hat{\lambda }}_{i}$ per group, sample variances, the proportion of zeros, and the ratio of variance and mean as estimation for overdispersion. The group sample sizes are unbalanced, ranging from 10 to 39; the estimated rates lie between 5.7 and 6.8, and the sample variances appear to be heteroscedastic, ranging from 21.1 to 52.5. The estimated overdispersion ranges from 3.1 to 9.1.

We used the Dunnett‐type contrast test to compare all age groups with the youngest group, aged 20–29. Table 4 shows the results for the eight methods examined in Section 5. The estimate of the contrast with corresponding 95% confidence intervals is given in the scale used within the technique. In addition, SE, test statistic, and *p*‐value are presented. In contrast, the largest estimated difference is 3−1 between the age groups 40–49 and 20–29, with a difference of −1.06 on the original scale. Even if none of the methods yield a significant *p*‐value (<0.05) for this contrast, differences in the estimated *p*‐values are noticeable, ranging from 0.40 to 0.90. The smallest *p*‐value for the contrast 3–1 with *p* = 0.4 is given by poi, which is a method assuming no overdispersion and often appeared too liberal in Section 5. Slightly larger values are provided by the methods het_log (*p* = 0.46) and het_sqrt (*p* = 0.60). The methods boot, hom, het, nb, and q_poi result in similar *p*‐values between 0.88 and 0.9 for contrast 3–1. Note that Tukey‐type and Grand‐Mean‐type contrasts also did not result in significant differences.

## 7 Discussion

Making inferences in count data is an ongoing challenge in statistical data sciences, especially when the sample sizes are small. An accurate Type I error rate control of the methods is a paramount requirement to avoid false positive conclusions. Until now, no statistical test has met this criterion in arbitrary count data distributional models. Most procedures rely on strict and specific distributional assumptions, such as data following a Poisson or Negative Binomial distribution and equal dispersion. In small samples, however, verifying these assumptions is impossible; therefore, gaining knowledge about the tests' behavior under model misspecifications is essential to prevent false‐positive decisions. In this paper, we investigate multiple contrast tests for over‐ and underdispersed count data under heteroscedasticity and group‐specific dispersion, examining their properties in small sample sizes. We introduced a general statistical model for count data to overcome strict model assumptions that allow for (i) under‐, equi‐, and overdispersion and (ii) group‐specific dispersions. This model covers all known models as special cases. We introduced a nonparametric bootstrap test to make inferences within this general setup. In a comprehensive simulation study, we examined its properties (Type I error rate control and power to detect selected alternatives) in various scenarios to evaluate and compare it with several competitors. The results of these simulations indicate that no single method consistently performs best across all investigated settings. However, the bootstrap test and the linear model with heteroscedastic variances perform well in most settings examined here. The linear model with heteroscedastic variances is slightly liberal in small sample sizes. The bootstrap test becomes slightly liberal when sample sizes and count rates are small, and somewhat conservative for small sample sizes combined with larger count rates. The GLM methods yielded partially liberal or conservative results regarding the Type I error rate. This is due to the assumptions underlying these methods, which are violated in some of the examined data situations. These assumptions refer to specific distributions and a constant dispersion parameter between the groups. Non‐fulfillment of the underlying assumptions is particularly problematic for small sample sizes. Consistent with the findings of O'Hara and Kotze (2010) and Warton (2018), our results indicate that data transformations are prone to an inflated Type I error in the examined data situations, particularly for positive and negative pairings.

Furthermore, a power analysis revealed only minor differences between the methods in terms of global power. Here, GLMs assuming a Poisson distribution and transformations with subsequent linear models were omitted from the examined methods due to high Type I errors in many settings, as liberal methods result in mistakenly high power.

All in all, even if no single method performs best in all investigated settings, the bootstrap test and the linear model with heteroscedastic variances perform well in most examined settings and are therefore most recommended. Referring to Heinze et al. (2024) regarding the phases of methodological research in biostatistics, we would classify our study as a Phase II study, as we included comparisons with other methods and provided example data analyses. As a next step, ideally, a neutral comparison study would compare our proposed nonparametric bootstrap test with others using simulations with a wider range of scenarios, distributions, and numbers of compared groups. In this simulation study, we investigated methods without covariate adjustments and flexible modeling. Model extensions of the bootstrap in these directions will be part of future research. See, for example, Becher et al. (2025) for covariate adjustments in bootstrap methods.

In practice, situations may arise where differentially weighting null hypotheses is desirable—for example, when prioritizing clinically relevant comparisons or incorporating prior evidence. In such cases, critical limits can no longer be derived from equi‐coordinate quantiles, requiring alternative approaches. While our current framework assumes equally weighted elementary hypotheses for compatibility with standard Dunnett/Tukey procedures, weighted testing strategies (e.g., via graphical approaches, see, e.g., Bretz et al. 2009; 2011); Maurer and Bretz (2016), or generally weighted procedures Benjamini and Hochberg (1997); Westfall and Young (1993); Dmitrienko et al. (2008); Meinshausen et al. (2009)) could offer flexibility for such scenarios. However, the multiple contrast tests considered rely on equi‐coordinate quantile estimation; extending this to accommodate user‐defined weights would necessitate methodological advancements, such as resampling‐adjusted critical limits or analytical approximations. This remains an important direction for future research.

As mentioned in the remark in Section 2, individual time frames as offset variables are a usual encounter in statistical practice. Ignoring them would bias the estimates and inference results; hence, the point and their variance estimators must be adjusted accordingly. Within Model 2,

$$X_{ik}∼F_{ik}\left(t_{ik}\lambda_{i},\sigma_{ik}^{2}\right),i=1,\text{…},a;\;k=1,\text{…},n_{i}.$$

The count rate is modeled as multiplicative; therefore, various weighing schemes exist to estimate the rates $\lambda_{i}$ and their variances. In the following, we propose two intuitive ways to estimate the count rates $\lambda_{i}$ and their variances without bias, namely

$$\begin{array}{c} {\hat{\lambda }}_{i}^{\left(u\right)}=\frac{1}{n_{i}}\sum_{k=1}^{n_{i}}\frac{X_{ik}}{t_{ik}}\;\;\text{or}\;\;{\hat{\lambda }}_{i}^{\left(w\right)}=\frac{1}{T_{i}}\sum_{k=1}^{n_{i}}X_{ik};\;T_{i}=\sum_{k=1}^{n_{i}}t_{ik}. \end{array}$$

The superscripts (u) and (w) refer to unweighted and weighted estimators, respectively. The unweighted estimator is nothing but the mean of the variables $Z_{ik}=\frac{X_{ik}}{t_{ik}}$. Since $t_{ik}>0$ by definition, numerical issues due to zero divisions cannot occur. In Model 2, every variable $X_{ik}$ may have different variances, so unbiased estimation is involved. Konietschke et al. (2019) developed unbiased variance estimators and show that the variance of ${\hat{\lambda }}_{i}^{\left(u\right)}$ can be estimated unbiasedly by

$$\begin{array}{c} {\hat{\sigma }}_{i}^{2,(u)}=\frac{1}{n_{i}\left(n_{i}-1\right)}\sum_{k=1}^{n_{i}}{\left(Z_{ik}-{\bar{Z}}_{ik}\right)}^{2},\;{\bar{Z}}_{ik}=\frac{1}{n_{i}}\sum_{k=1}^{n_{i}}Z_{ik}. \end{array}$$

Unbiased estimation of the variance of ${\hat{\lambda }}_{i}^{\left(w\right)}=\frac{1}{T_{i}}\sum_{k=1}^{n_{i}}X_{ik}$ is a bit tricky: Define the random variables ${\tilde{Z}}_{ik}=X_{ik}-t_{ik}{\hat{\lambda }}_{i}^{\left(w\right)}$, and note that $E({\tilde{Z}}_{ik})=0$ for all i=1,…,a, and $k=1,\text{…},n_{i}$. The variables ${\tilde{Z}}_{ik}$ describe the deviation of $X_{ik}$ to its estimated expectation. An unbiased moment‐based estimator can now be defined by considering the squared deviation from ${\tilde{Z}}_{ik}$ along with a bias correction, given by

$$\begin{array}{c} {\tilde{\sigma }}_{i}^{2,(w)}=\frac{1}{\left(1+K_{i}\right)T_{i}^{2}}\sum_{k=1}^{n_{i}}\frac{T_{i}}{\left(T_{i}-2t_{ik}\right)}{\tilde{Z}}_{ik}^{2},\;\text{where}\;K_{i}=\sum_{k=1}^{n_{i}}\frac{t_{ik}^{2}}{\left(T_{i}-2t_{ik}\right)T_{i}}. \end{array}$$

Routine calculations show that ${\tilde{\sigma }}_{i}^{2,(w)}$ is an unbiased estimator of $Var\left({\hat{\lambda }}_{i}^{\left(w\right)}\right)$. Once the estimators are adjusted for the offset individual varying time frames, their impact on the tests' quality is minor (Konietschke et al. 2019). Therefore, we discussed all methods in the simpler model for easier readability.

## Conflicts of Interest

The authors declare no conflicts of interest.

## Open Research Badges

This article has earned an Open Data badge for making publicly available the digitally‐shareable data necessary to reproduce the reported results. The data is available in the Supporting Information section.

This article has earned an open data badge “**Reproducible Research**” for making publicly available the code necessary to reproduce the reported results. The results reported in this article could fully be reproduced.

## Supporting information


**Supporting File 1:** bimj70098‐sup‐0001‐SuppMat.zip.

**Supporting File 2:** bimj70098‐sup‐0002‐SuppMat.pdf.

## Data Availability

The authors have nothing to report.

## Appendix A

**TABLE A1:** Characteristics of simulated distributions.

Distribution	λ	Dispersion parameter	Variance^1^	Proportion of zeros^2^	$\frac{\mathrm{Variance}}{\mathrm{Mean}}$
POI 1	1	—	1.0	0.37	1.0
POI 2	6	—	6.0	0.00	1.0
POI 3	10	—	10.0	0.00	1.0
CMP 1	1	0.2	1.6	0.46	1.6
CMP 2	1	0.5	1.3	0.43	1.3
CMP 3	1	2	0.7	0.28	0.7
CMP 4	6	0.2	19.7	0.06	3.3
CMP 5	6	0.5	10.8	0.02	1.8
CMP 6	6	2	3.1	0.00	0.5
CMP 7	10	0.2	38.3	0.02	3.8
CMP 8	10	0.5	18.9	0.00	1.9
CMP 9	10	2	5.1	0.00	0.5
NB 1	1	0.75	2.3	0.53	2.3
NB 2	1	2	1.5	0.44	1.5
NB 3	1	3	1.3	0.42	1.3
NB 4	1	5	1.2	0.40	1.2
NB 5	6	0.75	54.0	0.19	9.0
NB 6	6	2	24.0	0.06	4.0
NB 7	6	3	18.0	0.04	3.0
NB 8	6	5	13.2	0.02	2.2
NB 9	10	0.75	143.3	0.14	14.3
NB 10	10	2	60.0	0.03	6.0
NB 11	10	3	43.3	0.01	4.3
NB 12	10	5	30.0	0.00	3.0

1 Variance for Poisson given by λ, for Negative Binomial calculated with $V\left(X\right)=\mu +\frac{\mu^{2}}{size}$ and for Conway‐Maxwell Poisson estimated in a sample with n=100.000,

2 estimated in a sample of n=100.000
