# Supplementary figures and images for: Multiple Contrast Tests for Count Data: Small Sample Approximations and Their Limitations
Source: Biom J. 2025 Dec 7;67(6):e70098. doi: 10.1002/bimj.70098 (PMC12683215; doi:10.1002/bimj.70098)

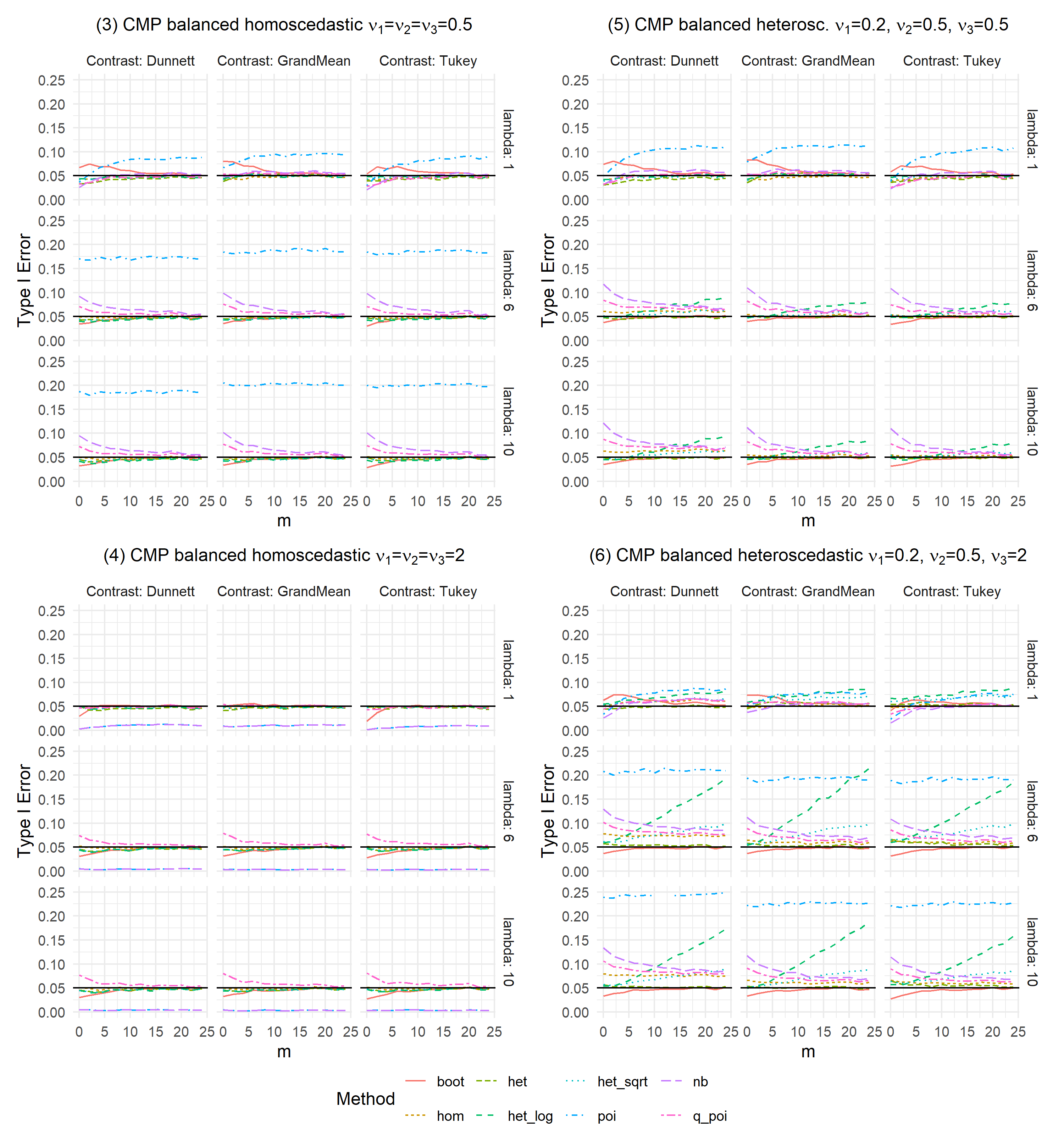

Supplement: Supplementary file 1 — Supporting File 1: bimj70098‐sup‐0001‐SuppMat.zip. [file BIMJ-67-e70098-s002.zip › MPigorsch_MCT_Count_Code/2_results/Results_Plots/Plot_CMP1_3.png]

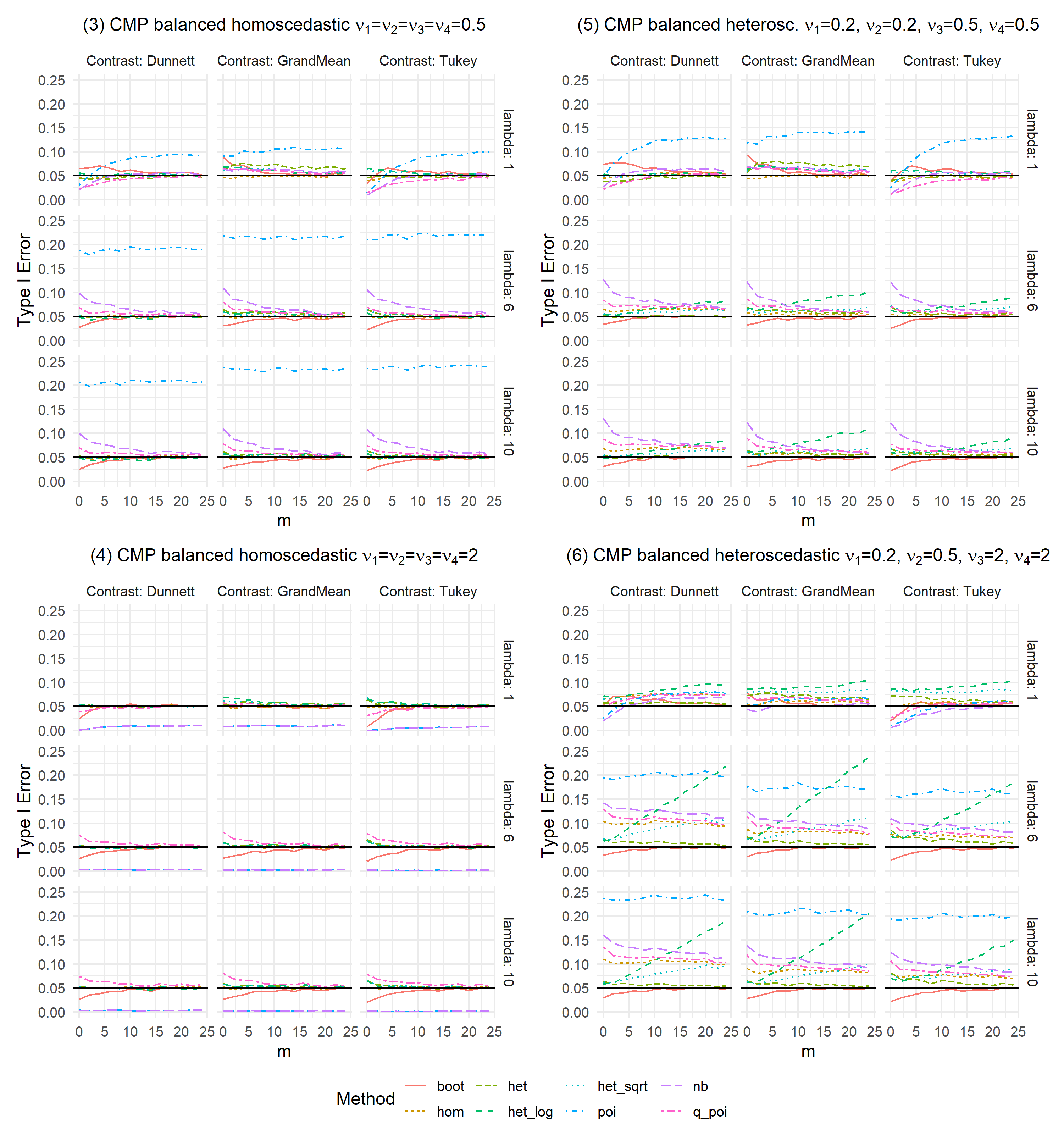

Supplement: Supplementary file 1 — Supporting File 1: bimj70098‐sup‐0001‐SuppMat.zip. [file BIMJ-67-e70098-s002.zip › MPigorsch_MCT_Count_Code/2_results/Results_Plots/Plot_CMP1_4.png]

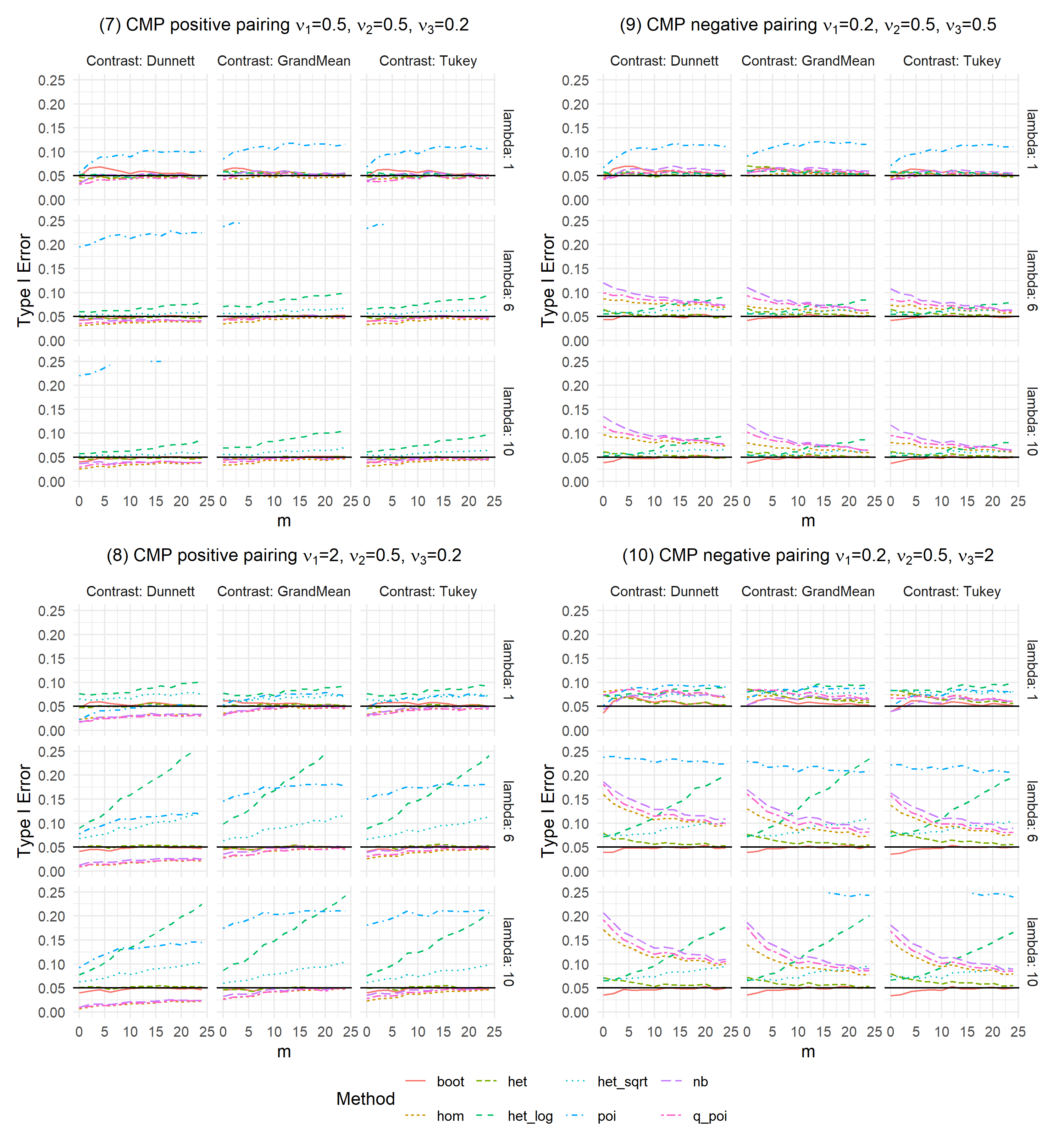

Supplement: Supplementary file 1 — Supporting File 1: bimj70098‐sup‐0001‐SuppMat.zip. [file BIMJ-67-e70098-s002.zip › MPigorsch_MCT_Count_Code/2_results/Results_Plots/Plot_CMP2_3.png]

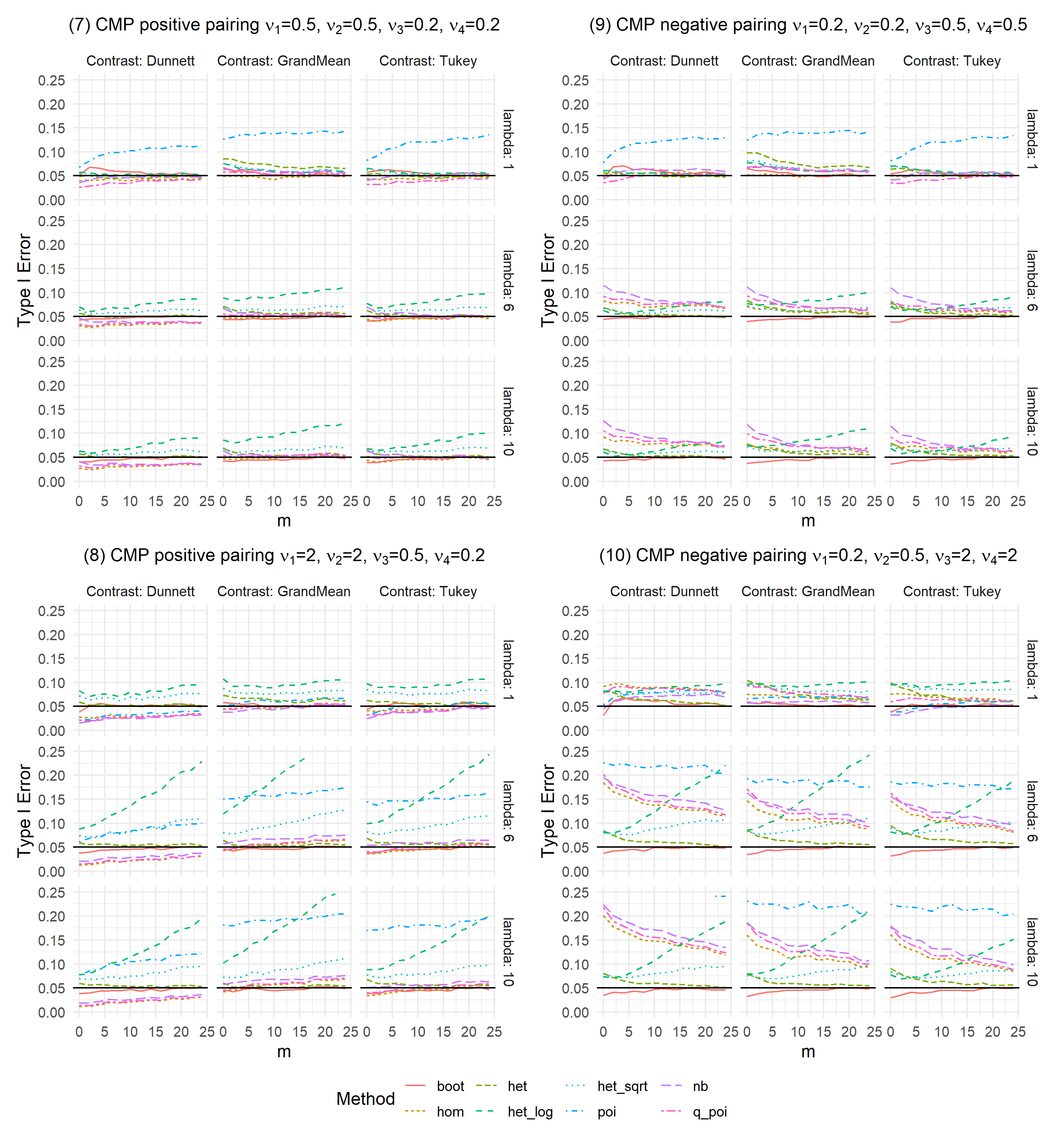

Supplement: Supplementary file 1 — Supporting File 1: bimj70098‐sup‐0001‐SuppMat.zip. [file BIMJ-67-e70098-s002.zip › MPigorsch_MCT_Count_Code/2_results/Results_Plots/Plot_CMP2_4.png]

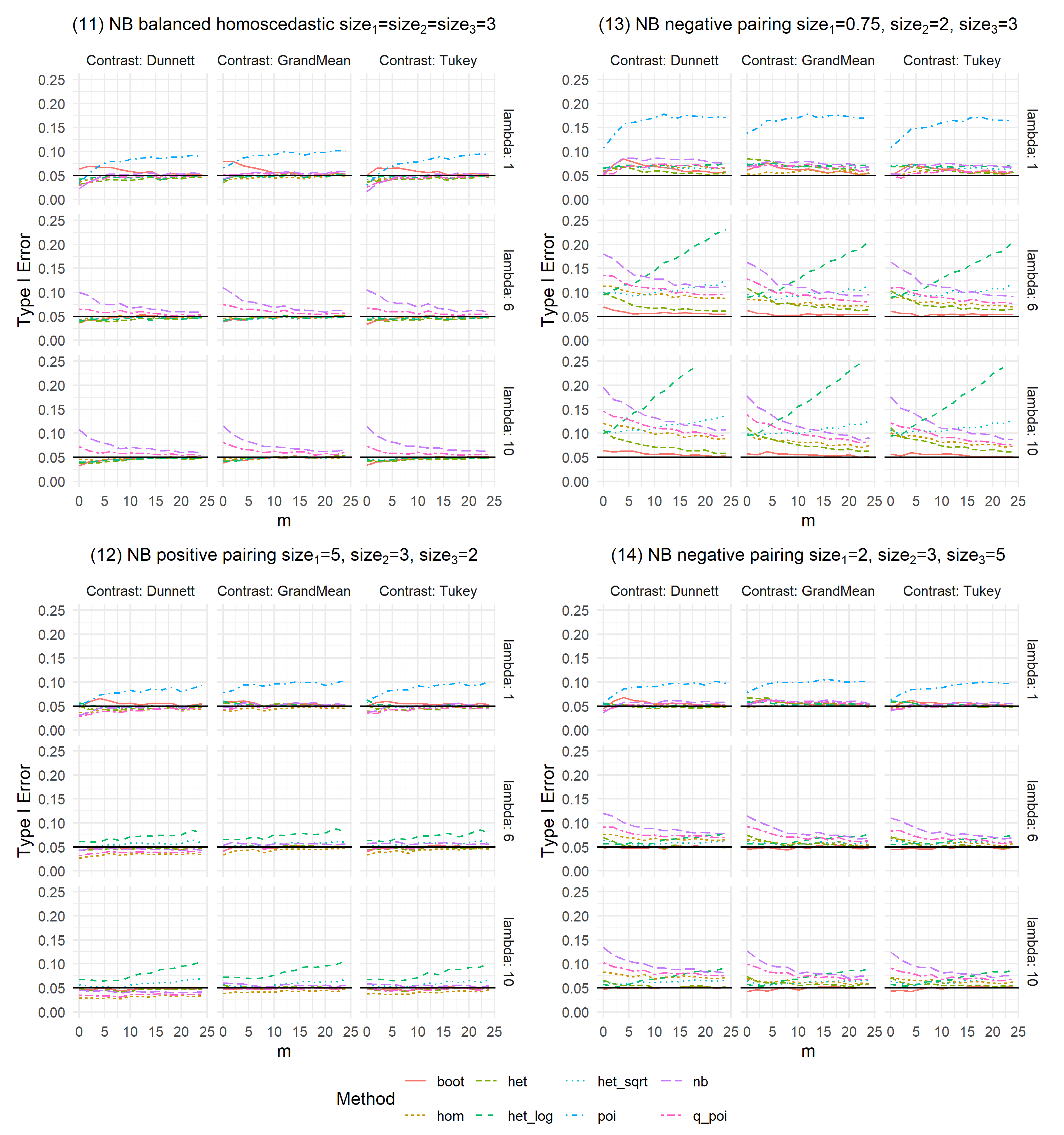

Supplement: Supplementary file 1 — Supporting File 1: bimj70098‐sup‐0001‐SuppMat.zip. [file BIMJ-67-e70098-s002.zip › MPigorsch_MCT_Count_Code/2_results/Results_Plots/Plot_NB_3.png]

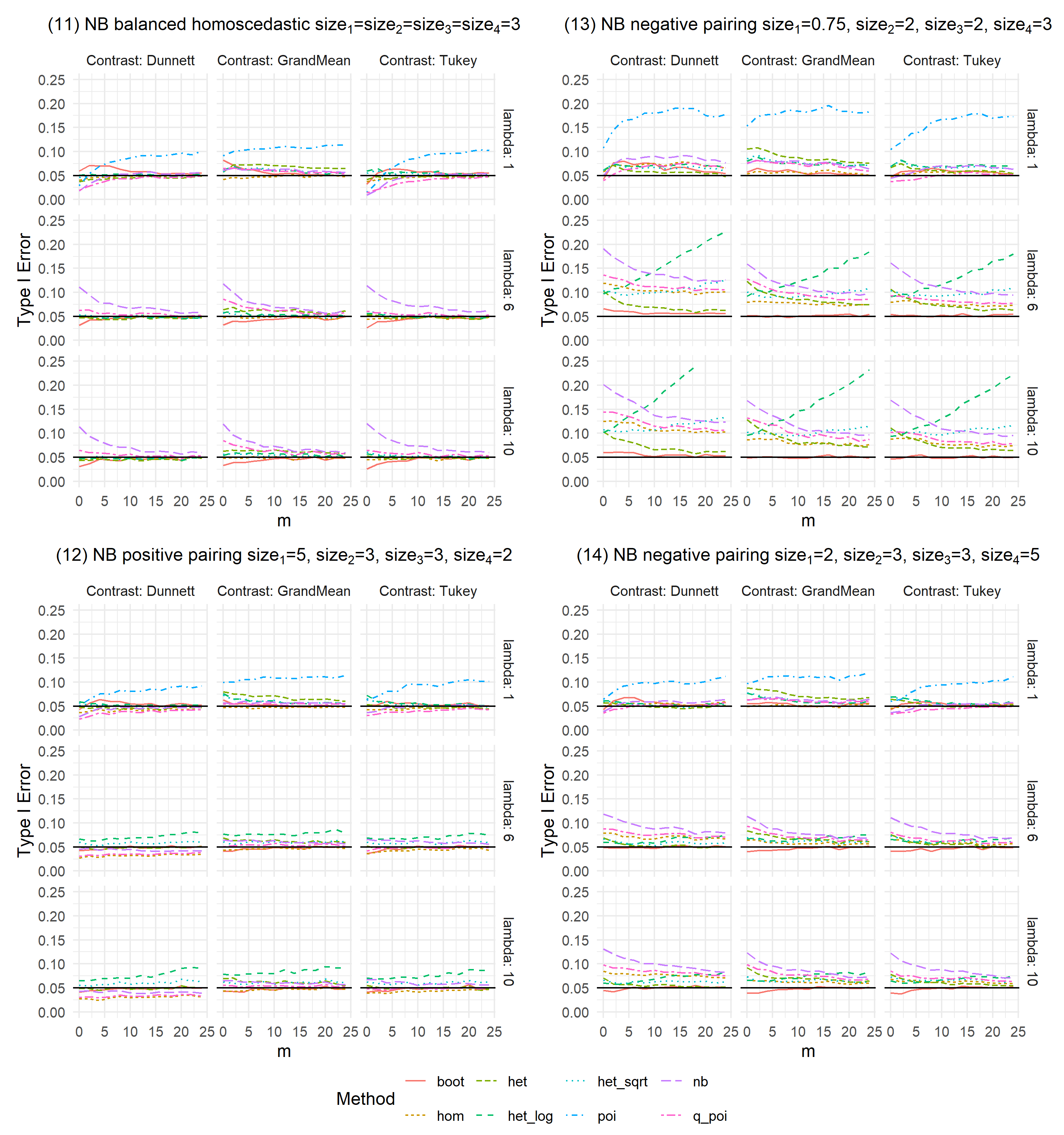

Supplement: Supplementary file 1 — Supporting File 1: bimj70098‐sup‐0001‐SuppMat.zip. [file BIMJ-67-e70098-s002.zip › MPigorsch_MCT_Count_Code/2_results/Results_Plots/Plot_NB_4.png]

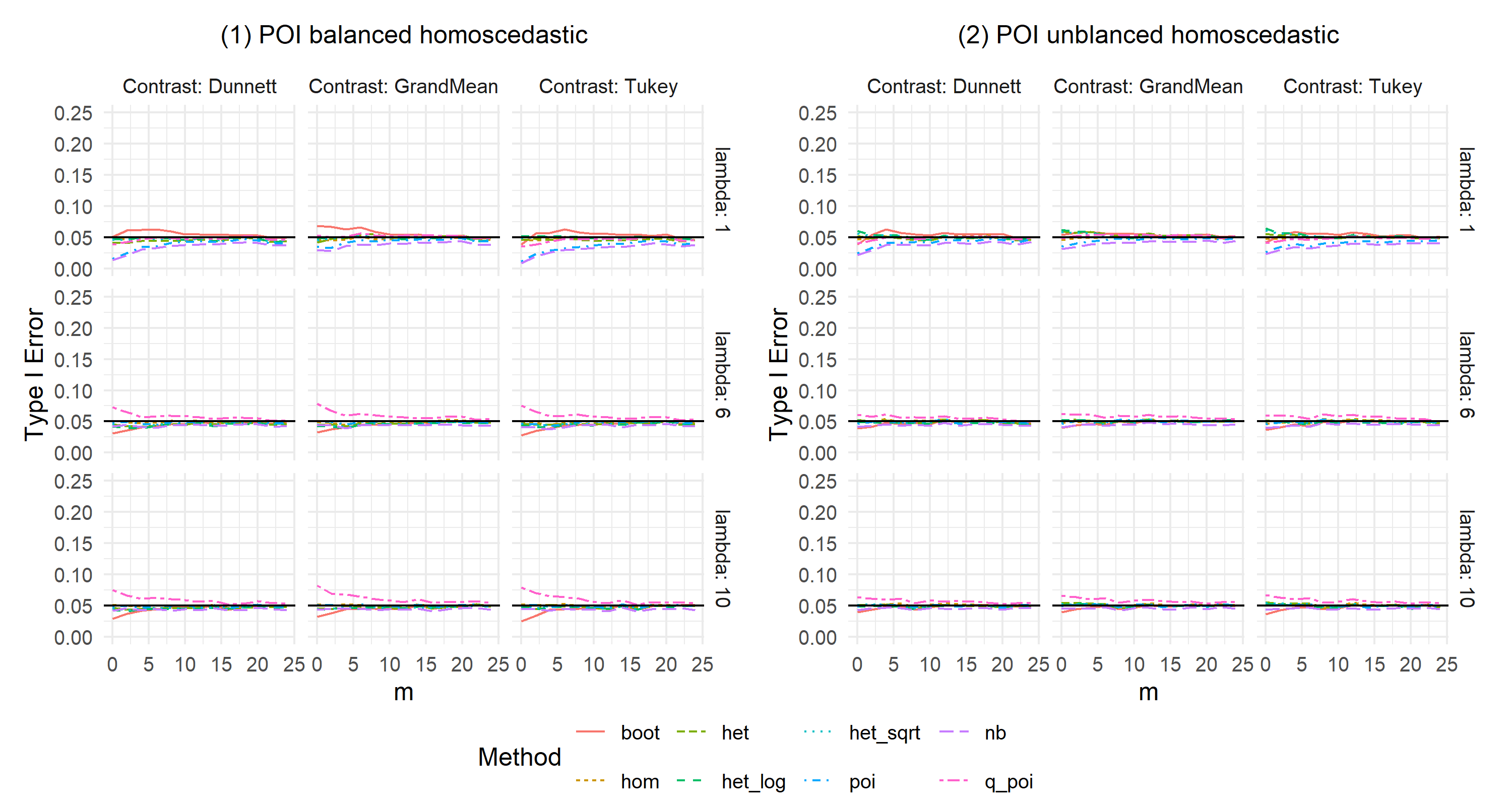

Supplement: Supplementary file 1 — Supporting File 1: bimj70098‐sup‐0001‐SuppMat.zip. [file BIMJ-67-e70098-s002.zip › MPigorsch_MCT_Count_Code/2_results/Results_Plots/Plot_POI_3.png]

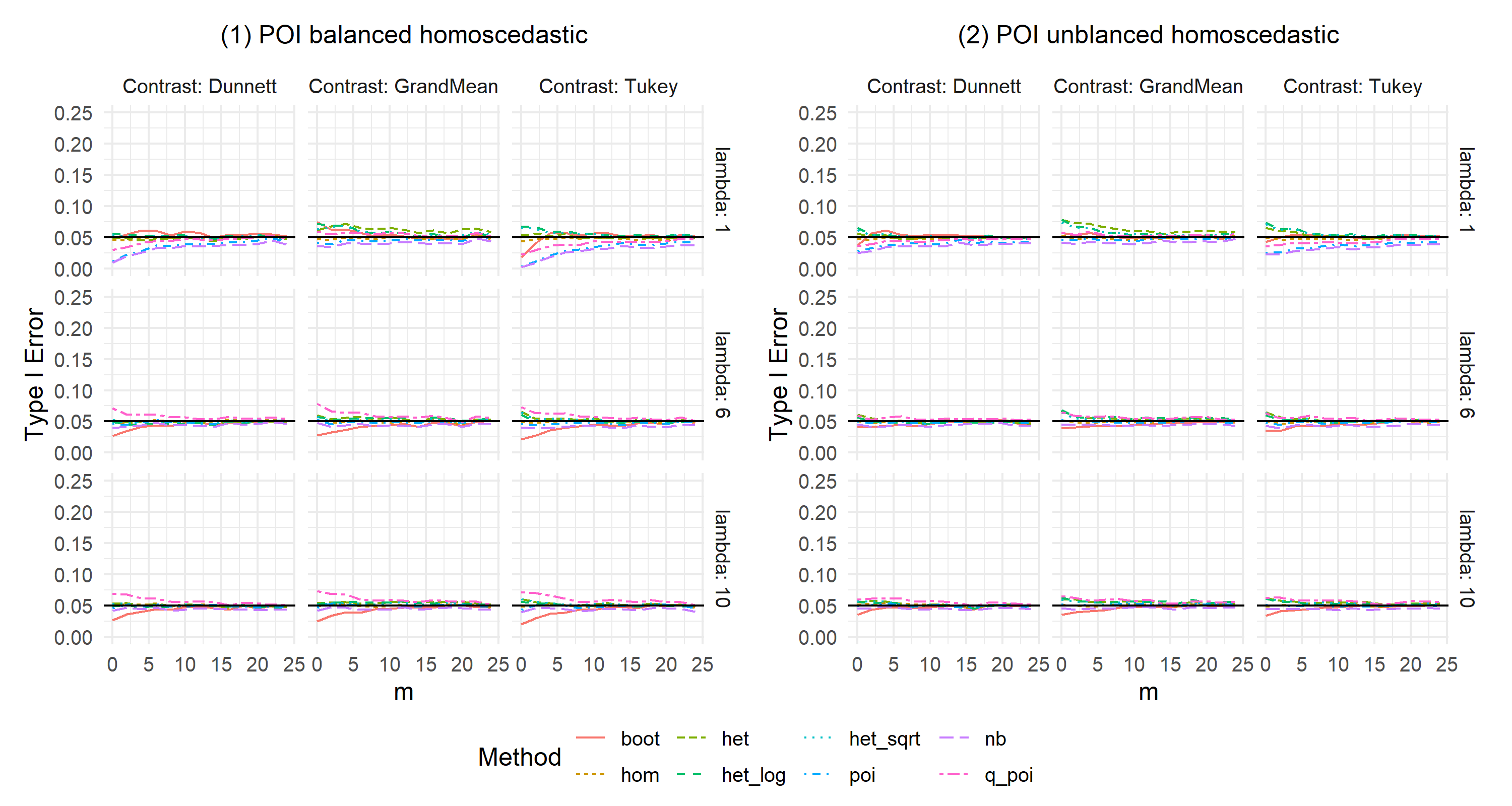

Supplement: Supplementary file 1 — Supporting File 1: bimj70098‐sup‐0001‐SuppMat.zip. [file BIMJ-67-e70098-s002.zip › MPigorsch_MCT_Count_Code/2_results/Results_Plots/Plot_POI_4.png]

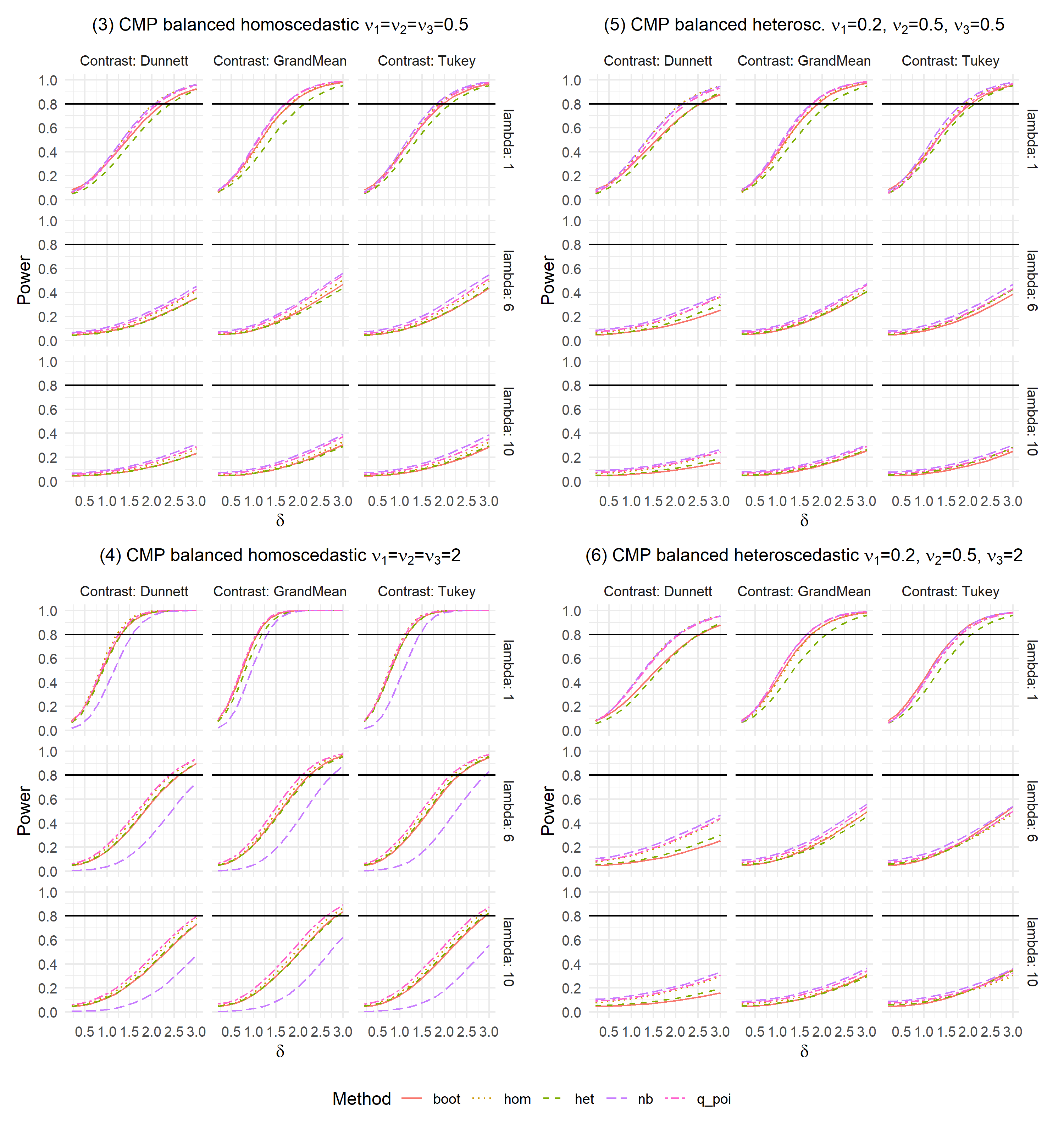

Supplement: Supplementary file 1 — Supporting File 1: bimj70098‐sup‐0001‐SuppMat.zip. [file BIMJ-67-e70098-s002.zip › MPigorsch_MCT_Count_Code/2_results/Results_Plots/PlotPower_CMP1_3.png]

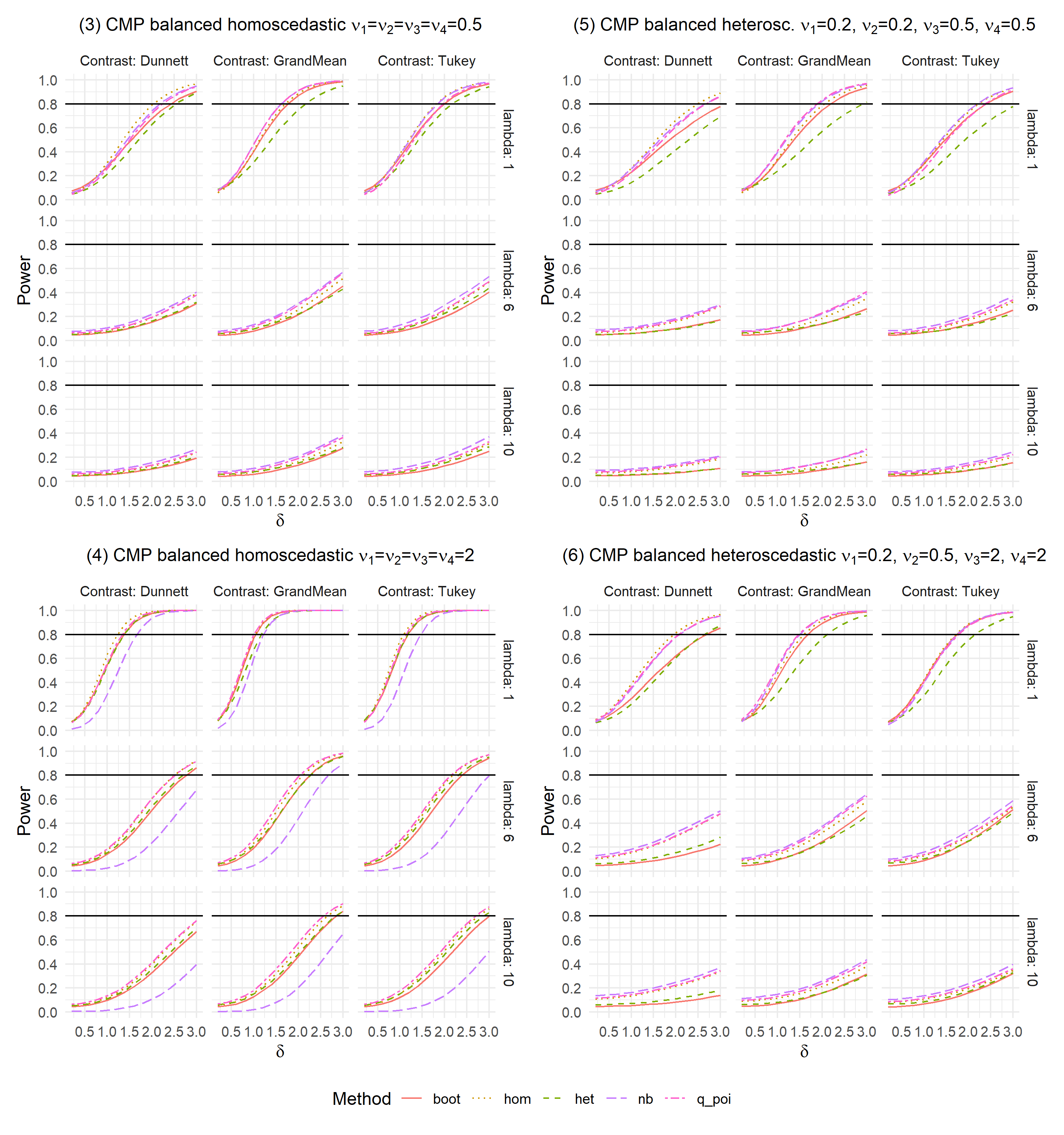

Supplement: Supplementary file 1 — Supporting File 1: bimj70098‐sup‐0001‐SuppMat.zip. [file BIMJ-67-e70098-s002.zip › MPigorsch_MCT_Count_Code/2_results/Results_Plots/PlotPower_CMP1_4.png]

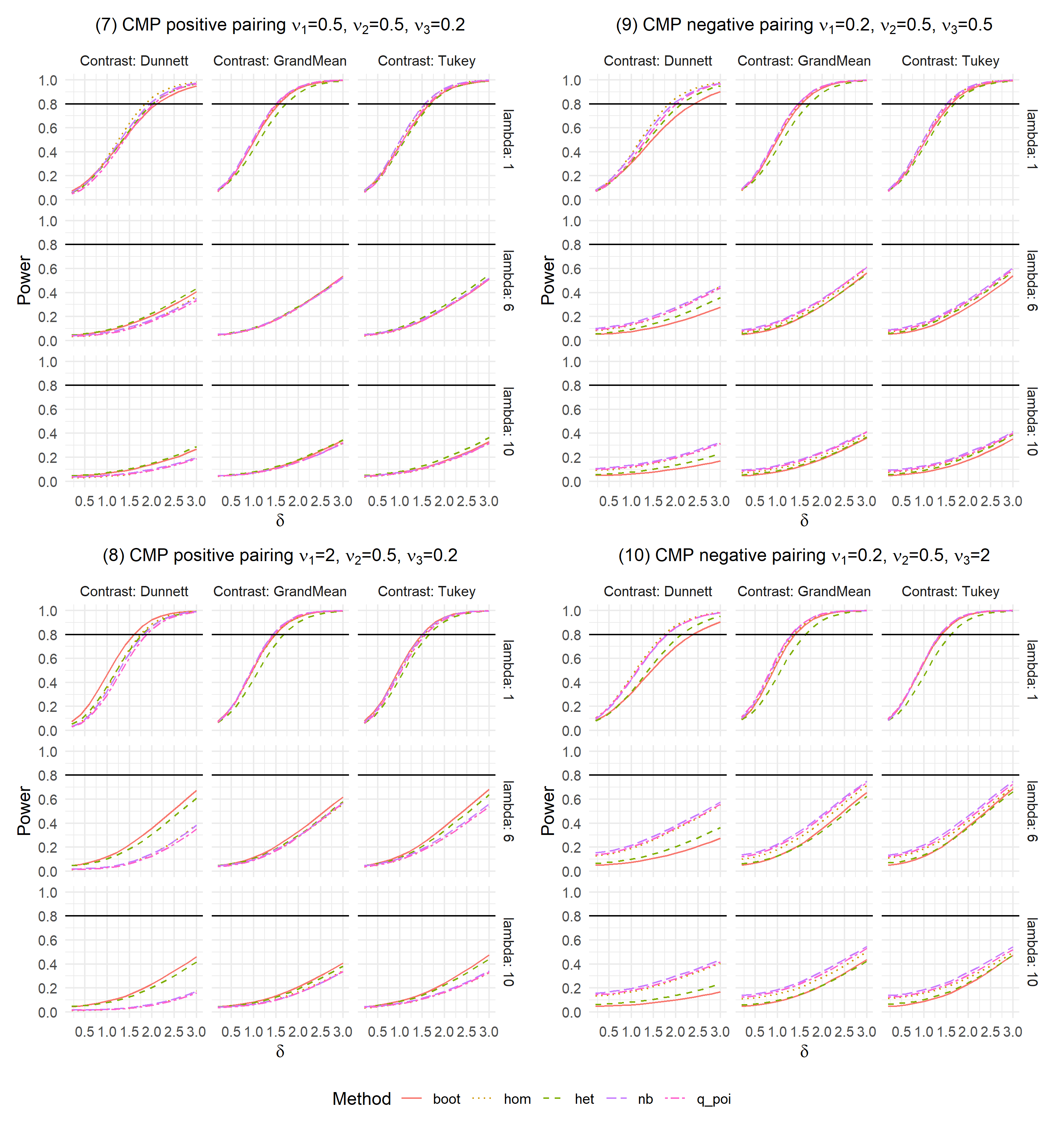

Supplement: Supplementary file 1 — Supporting File 1: bimj70098‐sup‐0001‐SuppMat.zip. [file BIMJ-67-e70098-s002.zip › MPigorsch_MCT_Count_Code/2_results/Results_Plots/PlotPower_CMP2_3.png]

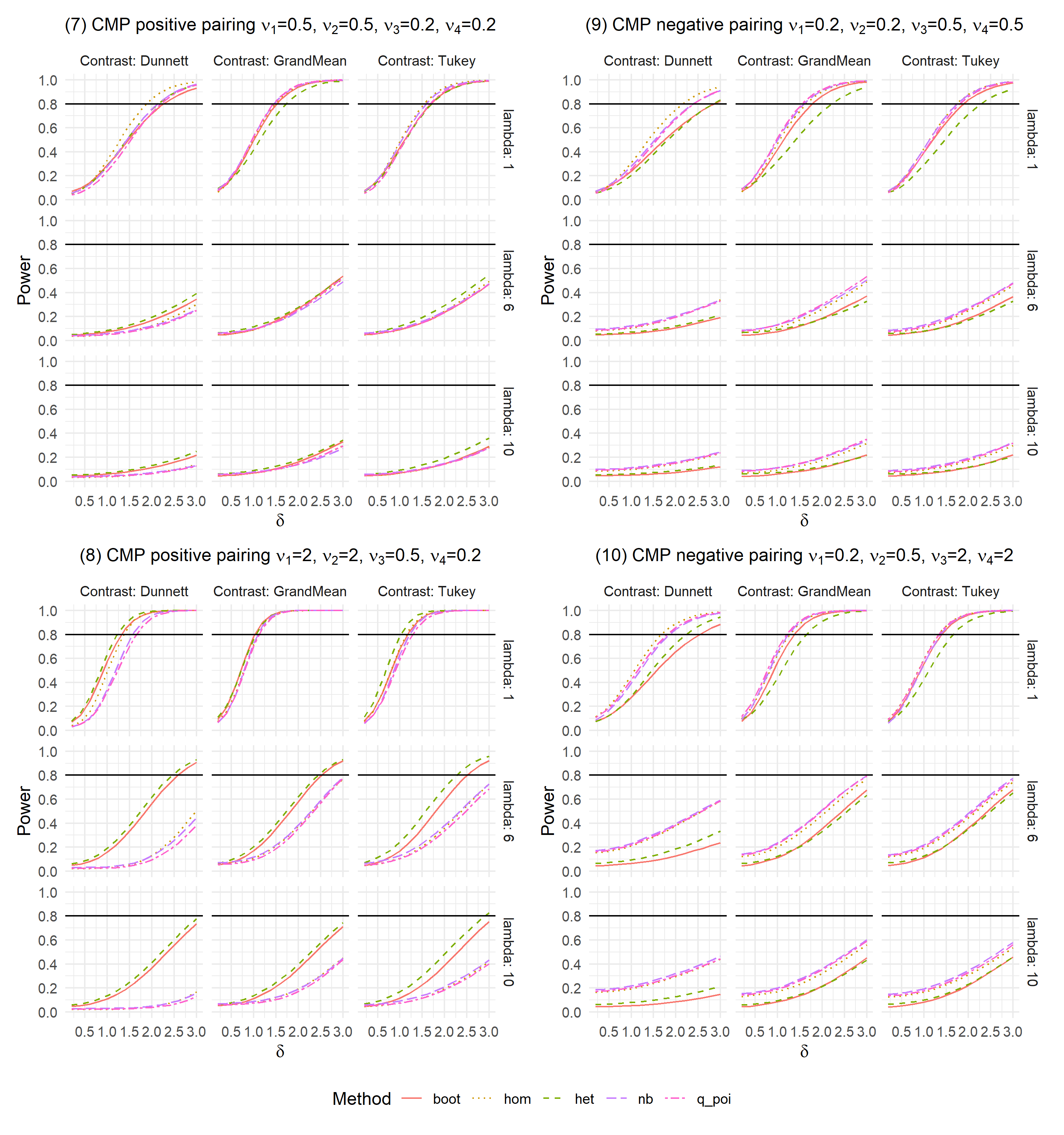

Supplement: Supplementary file 1 — Supporting File 1: bimj70098‐sup‐0001‐SuppMat.zip. [file BIMJ-67-e70098-s002.zip › MPigorsch_MCT_Count_Code/2_results/Results_Plots/PlotPower_CMP2_4.png]

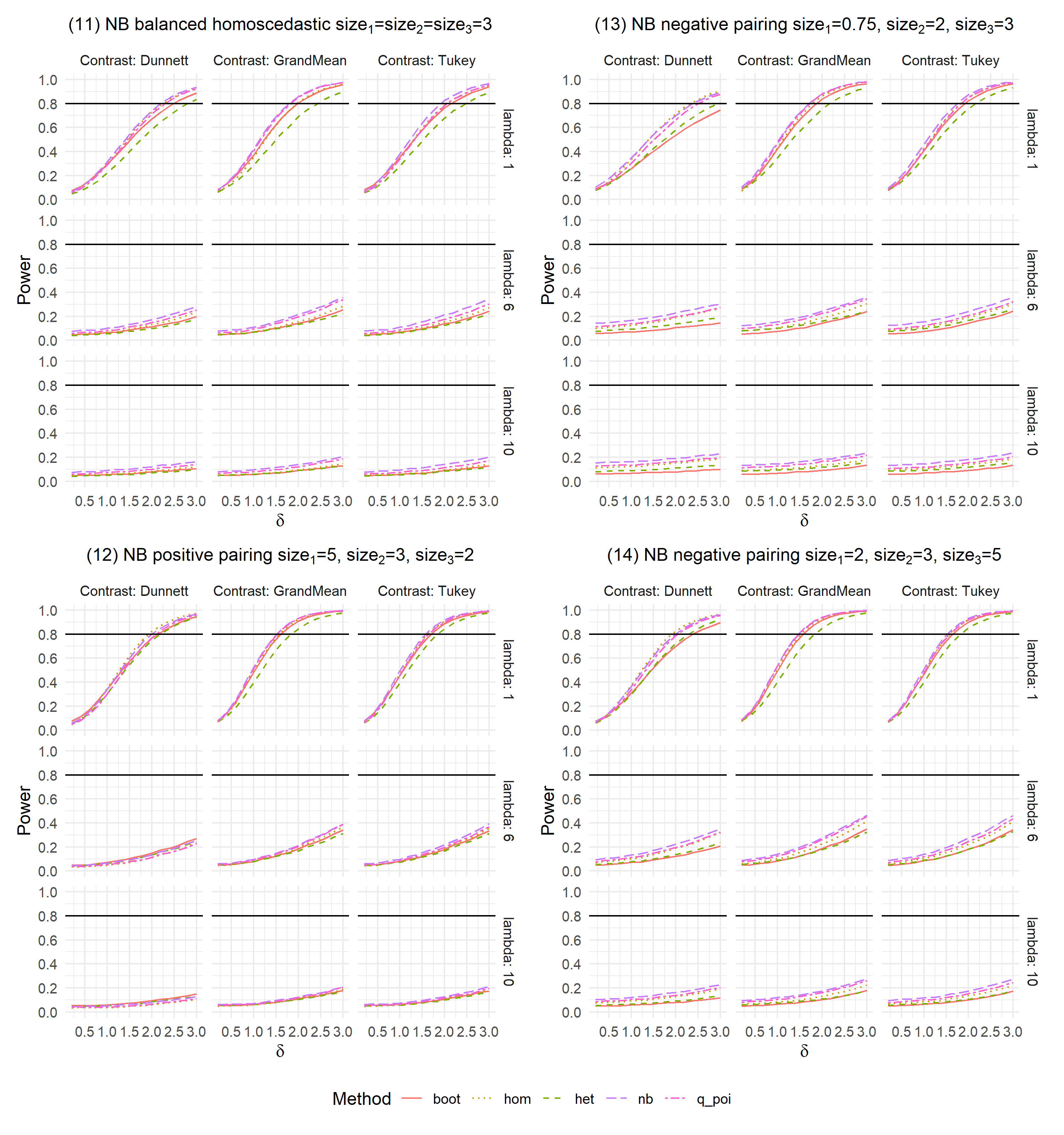

Supplement: Supplementary file 1 — Supporting File 1: bimj70098‐sup‐0001‐SuppMat.zip. [file BIMJ-67-e70098-s002.zip › MPigorsch_MCT_Count_Code/2_results/Results_Plots/PlotPower_NB_3.png]

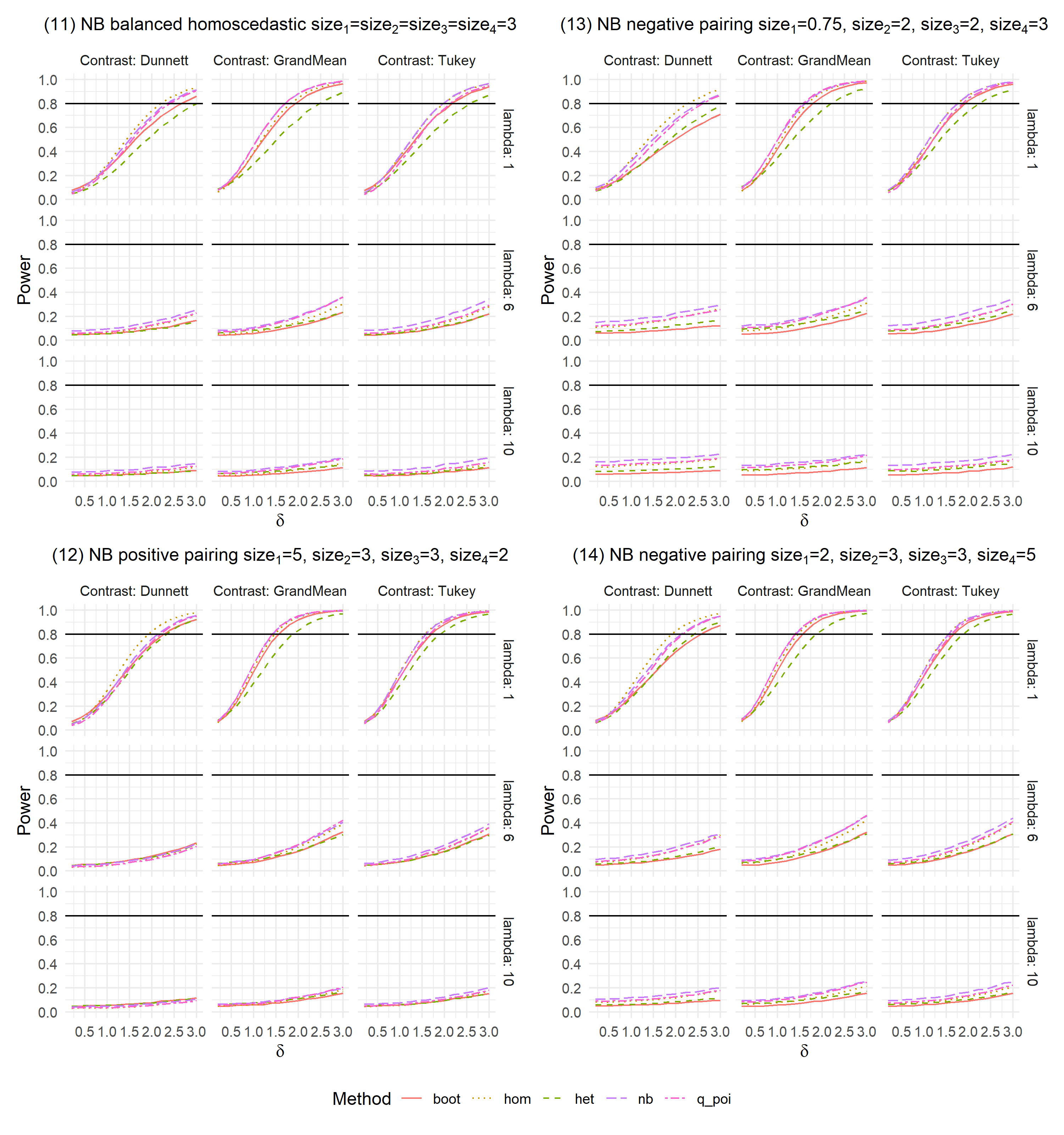

Supplement: Supplementary file 1 — Supporting File 1: bimj70098‐sup‐0001‐SuppMat.zip. [file BIMJ-67-e70098-s002.zip › MPigorsch_MCT_Count_Code/2_results/Results_Plots/PlotPower_NB_4.png]

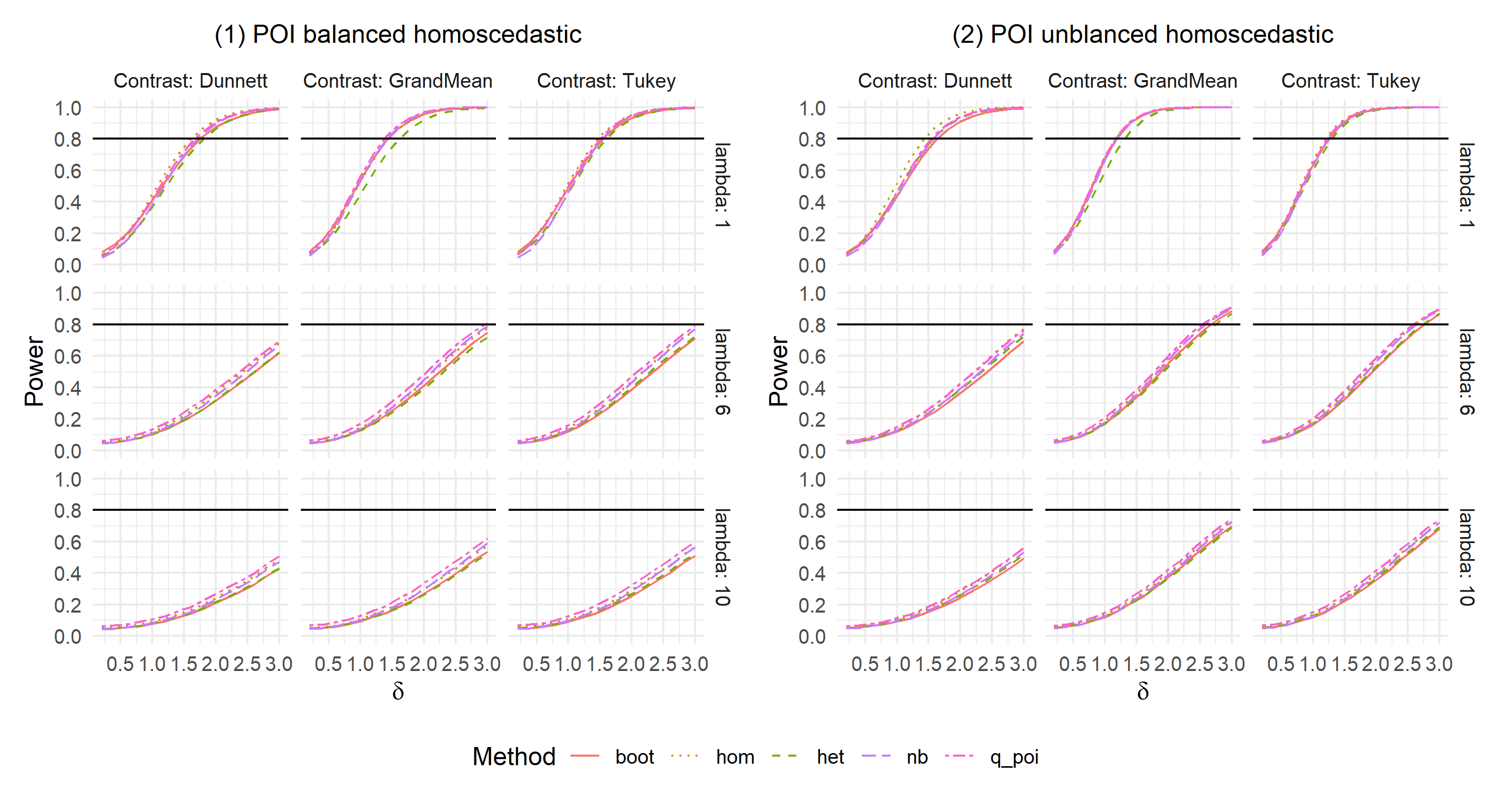

Supplement: Supplementary file 1 — Supporting File 1: bimj70098‐sup‐0001‐SuppMat.zip. [file BIMJ-67-e70098-s002.zip › MPigorsch_MCT_Count_Code/2_results/Results_Plots/PlotPower_POI_3.png]

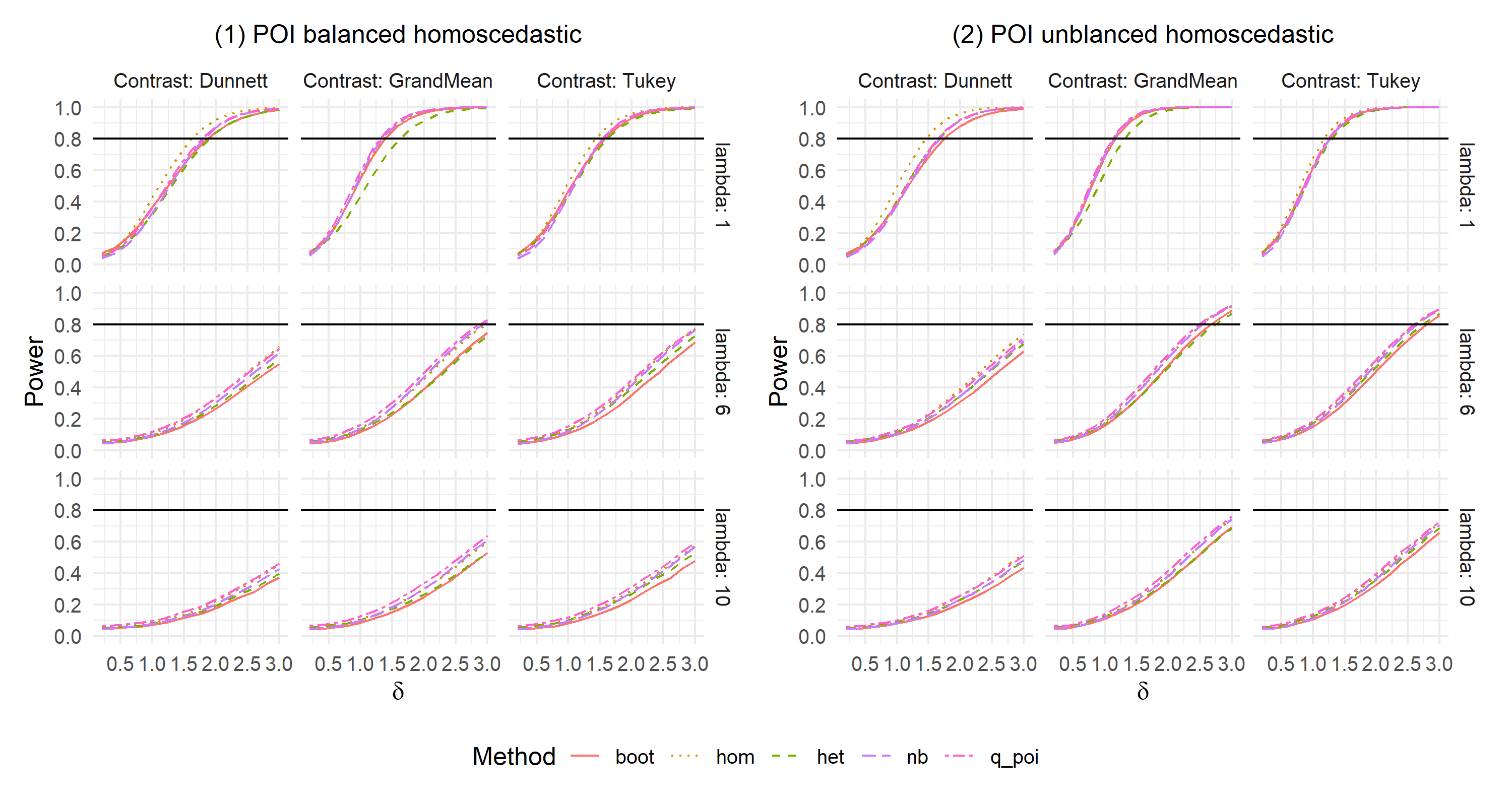

Supplement: Supplementary file 1 — Supporting File 1: bimj70098‐sup‐0001‐SuppMat.zip. [file BIMJ-67-e70098-s002.zip › MPigorsch_MCT_Count_Code/2_results/Results_Plots/PlotPower_POI_4.png]

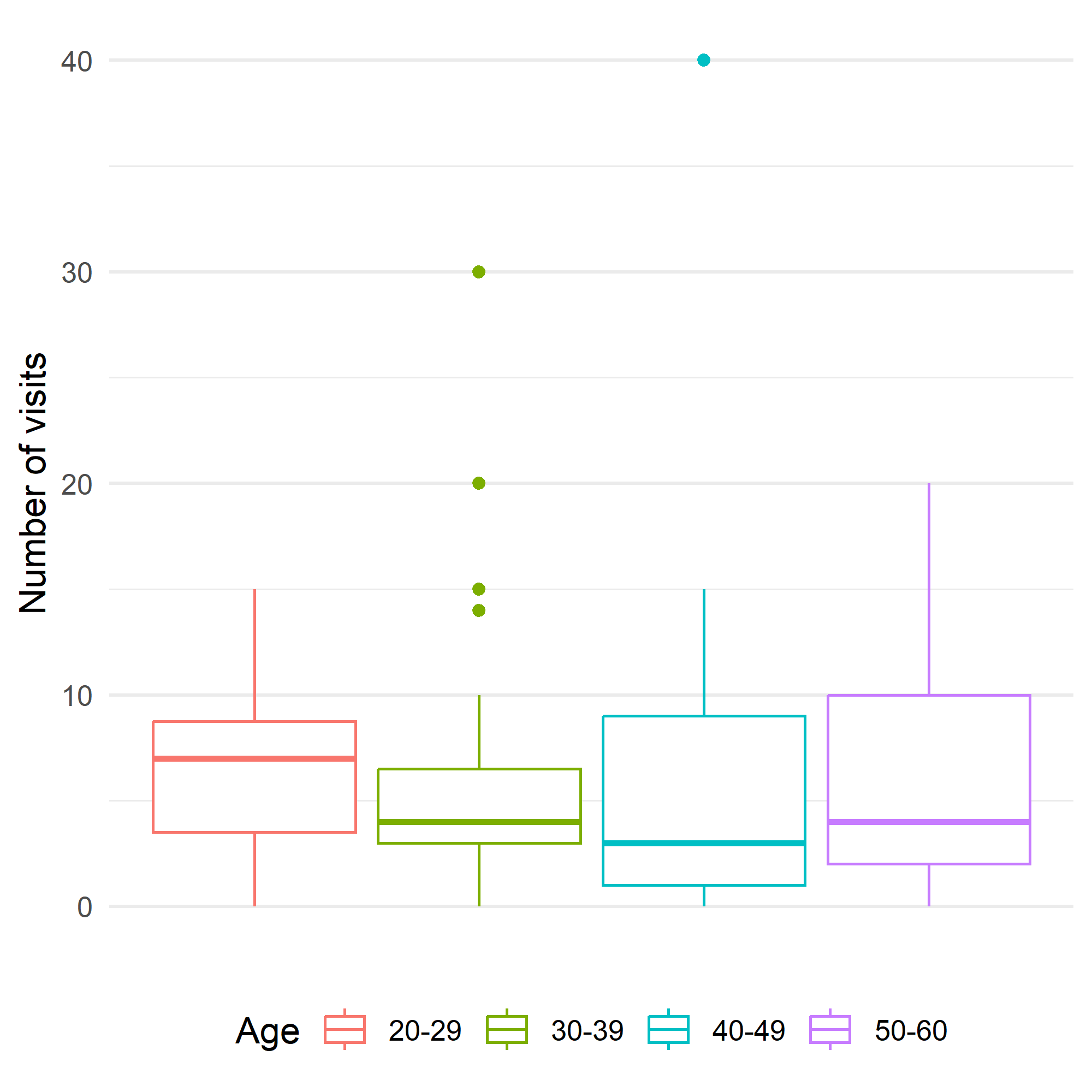

Supplement: Supplementary file 1 — Supporting File 1: bimj70098‐sup‐0001‐SuppMat.zip. [file BIMJ-67-e70098-s002.zip › MPigorsch_MCT_Count_Code/3_application/Application_Boxplot.png]
