# Supplementary material for: Multiple Contrast Tests for Count Data: Small Sample Approximations and Their Limitations
Source: Biom J. 2025 Dec 7;67(6):e70098. doi: 10.1002/bimj.70098 (PMC12683215; doi:10.1002/bimj.70098)
Supplement: Supplementary file 2 — Supporting File 2: bimj70098‐sup‐0002‐SuppMat.pdf. [file BIMJ-67-e70098-s001.pdf]

Supplementary material for ”Multiple contrast tests for possible overdispersed count data: small sample approximations”

Mareen Pigorsch[*,1], **Ludwig A. Hothorn** [2], and **Frank Konietschke** [1]

[1] Charité – Universitätsmedizin Berlin, Institute of Biometry and Clinical Epidemiology, Charitéplatz 1, 10117 Berlin, Germany

[2] retired from Leibniz University Hannover, Lauenau, Germany

*Corresponding author: e-mail: mareen.pigorsch@charite.de

1 Simulation results for different power types: global, any-pairs, all-pairs

Powertype	boot	hom	het	nb	q_poi	λ	Setting	Distrib.
global	0.44	0.51	0.46	0.50	0.52	6	1	POI
any	0.43	0.50	0.45	0.49	0.51	6	1	POI
all	0.11	0.12	0.11	0.13	0.15	6	1	POI
global	0.53	0.61	0.60	0.59	0.62	6	2	POI
any	0.52	0.61	0.59	0.58	0.60	6	2	POI
all	0.14	0.15	0.16	0.17	0.19	6	2	POI
global	0.25	0.29	0.26	0.34	0.31	6	3	CMP
any	0.24	0.29	0.25	0.32	0.29	6	3	CMP
all	0.05	0.04	0.04	0.09	0.07	6	3	CMP
global	0.74	0.80	0.76	0.48	0.82	6	4	CMP
any	0.73	0.80	0.76	0.48	0.81	6	4	CMP
all	0.26	0.28	0.27	0.08	0.33	6	4	CMP
global	0.17	0.24	0.22	0.27	0.24	6	5	CMP
any	0.16	0.22	0.21	0.24	0.22	6	5	CMP
all	0.04	0.05	0.05	0.08	0.06	6	5	CMP
global	0.21	0.42	0.34	0.45	0.39	6	6	CMP
any	0.20	0.39	0.34	0.41	0.36	6	6	CMP
all	0.08	0.17	0.15	0.20	0.17	6	6	CMP
global	0.24	0.19	0.24	0.23	0.20	6	7	CMP
any	0.23	0.19	0.23	0.23	0.20	6	7	CMP
all	0.04	0.02	0.03	0.04	0.03	6	7	CMP
global	0.43	0.26	0.35	0.34	0.30	6	8	CMP
any	0.42	0.26	0.35	0.34	0.30	6	8	CMP
all	0.08	0.01	0.05	0.04	0.03	6	8	CMP
global	0.19	0.31	0.28	0.34	0.29	6	9	CMP
any	0.18	0.29	0.28	0.30	0.27	6	9	CMP
all	0.06	0.07	0.08	0.11	0.08	6	9	CMP
global	0.21	0.56	0.38	0.58	0.53	6	10	CMP
any	0.21	0.52	0.37	0.52	0.48	6	10	CMP
all	0.08	0.23	0.17	0.26	0.23	6	10	CMP
global	0.15	0.18	0.14	0.23	0.19	6	11	NB
any	0.14	0.17	0.13	0.21	0.18	6	11	NB
all	0.02	0.01	0.01	0.04	0.03	6	11	NB
global	0.19	0.13	0.18	0.20	0.15	6	12	NB
any	0.18	0.13	0.17	0.19	0.14	6	12	NB
all	0.03	0.01	0.03	0.03	0.02	6	12	NB
global	0.13	0.22	0.21	0.28	0.22	6	13	NB
any	0.12	0.21	0.20	0.26	0.21	6	13	NB
all	0.05	0.05	0.07	0.11	0.07	6	13	NB
global	0.17	0.24	0.25	0.29	0.24	6	14	NB
any	0.16	0.23	0.24	0.27	0.22	6	14	NB
all	0.04	0.04	0.05	0.09	0.06	6	14	NB

Table S1 Power-types for contrast Dunnett with $\tilde{\lambda}_1 = \tilde{\lambda}_2 = \lambda, \tilde{\lambda}_3 = \lambda + 1.5, \tilde{\lambda}_4 = \lambda + 2.5$ and $\lambda = 6$, resulting in one true and two false null hypotheses.

Powertype	boot	hom	het	nb	q_poi	λ	Setting	Distrib.
global	0.65	0.69	0.68	0.72	0.73	6	1	POI
any	0.65	0.69	0.68	0.72	0.73	6	1	POI
all	0.10	0.08	0.09	0.12	0.14	6	1	POI
global	0.71	0.77	0.77	0.78	0.79	6	2	POI
any	0.71	0.77	0.77	0.78	0.79	6	2	POI
all	0.14	0.12	0.15	0.16	0.18	6	2	POI
global	0.39	0.42	0.41	0.50	0.46	6	3	CMP
any	0.39	0.42	0.41	0.50	0.46	6	3	CMP
all	0.04	0.02	0.03	0.08	0.05	6	3	CMP
global	0.92	0.95	0.93	0.76	0.96	6	4	CMP
any	0.92	0.95	0.93	0.76	0.96	6	4	CMP
all	0.25	0.24	0.26	0.08	0.33	6	4	CMP
global	0.24	0.32	0.28	0.38	0.35	6	5	CMP
any	0.24	0.32	0.28	0.38	0.35	6	5	CMP
all	0.03	0.02	0.03	0.07	0.04	6	5	CMP
global	0.28	0.51	0.40	0.57	0.52	6	6	CMP
any	0.28	0.51	0.40	0.57	0.52	6	6	CMP
all	0.07	0.12	0.11	0.19	0.15	6	6	CMP
global	0.41	0.28	0.44	0.35	0.31	6	7	CMP
any	0.41	0.28	0.44	0.35	0.31	6	7	CMP
all	0.03	0.01	0.03	0.04	0.02	6	7	CMP
global	0.92	0.41	0.93	0.54	0.47	6	8	CMP
any	0.92	0.41	0.93	0.54	0.47	6	8	CMP
all	0.08	0.01	0.05	0.04	0.02	6	8	CMP
global	0.25	0.37	0.34	0.43	0.39	6	9	CMP
any	0.25	0.37	0.34	0.43	0.39	6	9	CMP
all	0.04	0.03	0.06	0.10	0.06	6	9	CMP
global	0.28	0.61	0.43	0.64	0.61	6	10	CMP
any	0.28	0.61	0.43	0.64	0.61	6	10	CMP
all	0.08	0.17	0.14	0.25	0.22	6	10	CMP
global	0.22	0.23	0.22	0.32	0.27	6	11	NB
any	0.22	0.23	0.22	0.32	0.27	6	11	NB
all	0.01	0.00	0.01	0.03	0.01	6	11	NB
global	0.30	0.19	0.29	0.30	0.22	6	12	NB
any	0.30	0.19	0.29	0.30	0.22	6	12	NB
all	0.02	0.00	0.02	0.02	0.01	6	12	NB
global	0.15	0.26	0.25	0.34	0.27	6	13	NB
any	0.15	0.26	0.25	0.34	0.27	6	13	NB
all	0.04	0.02	0.05	0.09	0.05	6	13	NB
global	0.23	0.30	0.30	0.38	0.31	6	14	NB
any	0.23	0.30	0.30	0.38	0.31	6	14	NB
all	0.04	0.01	0.04	0.07	0.04	6	14	NB

Table S2 Power-types for contrast Dunnett, $\tilde{\lambda}_1 = \lambda$, $\tilde{\lambda}_2 = \lambda + 1.5$, $\tilde{\lambda}_3 = \lambda + 2.5$, $\tilde{\lambda}_4 = \lambda + 3$ and $\lambda = 6$, all null hypotheses are false.

Powertype	boot	hom	het	nb	q_poi	λ	Setting	Distrib.
global	0.48	0.56	0.53	0.56	0.59	6	1	POI
any	0.48	0.56	0.53	0.56	0.59	6	1	POI
all	0.00	0.00	0.00	0.00	0.00	6	1	POI
global	0.66	0.73	0.74	0.73	0.74	6	2	POI
any	0.66	0.73	0.74	0.73	0.74	6	2	POI
all	0.00	0.00	0.00	0.00	0.00	6	2	POI
global	0.26	0.32	0.30	0.39	0.35	6	3	CMP
any	0.26	0.32	0.30	0.39	0.34	6	3	CMP
all	0.00	0.00	0.00	0.00	0.00	6	3	CMP
global	0.79	0.85	0.83	0.50	0.88	6	4	CMP
any	0.79	0.85	0.83	0.50	0.88	6	4	CMP
all	0.00	0.00	0.00	0.00	0.00	6	4	CMP
global	0.19	0.27	0.28	0.31	0.27	6	5	CMP
any	0.19	0.26	0.28	0.30	0.26	6	5	CMP
all	0.00	0.00	0.00	0.00	0.00	6	5	CMP
global	0.31	0.46	0.53	0.51	0.43	6	6	CMP
any	0.31	0.45	0.53	0.50	0.43	6	6	CMP
all	0.00	0.00	0.00	0.00	0.00	6	6	CMP
global	0.30	0.23	0.29	0.30	0.26	6	7	CMP
any	0.30	0.23	0.29	0.30	0.26	6	7	CMP
all	0.00	0.00	0.00	0.00	0.00	6	7	CMP
global	0.48	0.29	0.39	0.39	0.35	6	8	CMP
any	0.48	0.29	0.38	0.39	0.35	6	8	CMP
all	0.00	0.00	0.00	0.00	0.00	6	8	CMP
global	0.26	0.38	0.40	0.42	0.37	6	9	CMP
any	0.26	0.37	0.40	0.42	0.36	6	9	CMP
all	0.00	0.00	0.00	0.00	0.00	6	9	CMP
global	0.45	0.67	0.67	0.71	0.65	6	10	CMP
any	0.45	0.67	0.67	0.70	0.64	6	10	CMP
all	0.00	0.00	0.00	0.00	0.00	6	10	CMP
global	0.16	0.18	0.16	0.26	0.21	6	11	NB
any	0.16	0.18	0.16	0.26	0.20	6	11	NB
all	0.00	0.00	0.00	0.00	0.00	6	11	NB
global	0.22	0.17	0.22	0.27	0.19	6	12	NB
any	0.22	0.17	0.21	0.27	0.19	6	12	NB
all	0.00	0.00	0.00	0.00	0.00	6	12	NB
global	0.17	0.22	0.29	0.30	0.21	6	13	NB
any	0.17	0.21	0.29	0.29	0.20	6	13	NB
all	0.00	0.00	0.00	0.00	0.00	6	13	NB
global	0.24	0.27	0.35	0.36	0.28	6	14	NB
any	0.24	0.27	0.35	0.35	0.28	6	14	NB
all	0.00	0.00	0.00	0.00	0.00	6	14	NB

Table S3 Power-types for contrast Tukey, $\tilde{\lambda}_1 = \tilde{\lambda}_2 = \lambda$, $\tilde{\lambda}_3 = \lambda + 1.5$, $\tilde{\lambda}_4 = \lambda + 2.5$ and $\lambda = 6$, resulting in one true and five false null hypotheses.

Powertype	boot	hom	het	nb	q_poi	λ	Setting	Distrib.
global	0.54	0.61	0.61	0.63	0.66	6	1	POI
any	0.54	0.61	0.61	0.63	0.66	6	1	POI
all	0.00	0.00	0.00	0.00	0.00	6	1	POI
global	0.63	0.69	0.71	0.70	0.72	6	2	POI
any	0.63	0.69	0.71	0.70	0.72	6	2	POI
all	0.00	0.00	0.00	0.00	0.00	6	2	POI
global	0.30	0.34	0.35	0.44	0.39	6	3	CMP
any	0.30	0.34	0.35	0.44	0.39	6	3	CMP
all	0.00	0.00	0.00	0.00	0.00	6	3	CMP
global	0.85	0.91	0.90	0.63	0.93	6	4	CMP
any	0.85	0.91	0.90	0.63	0.93	6	4	CMP
all	0.00	0.00	0.00	0.00	0.00	6	4	CMP
global	0.18	0.25	0.24	0.33	0.28	6	5	CMP
any	0.18	0.25	0.24	0.33	0.28	6	5	CMP
all	0.00	0.00	0.00	0.00	0.00	6	5	CMP
global	0.24	0.45	0.39	0.51	0.45	6	6	CMP
any	0.24	0.45	0.39	0.51	0.45	6	6	CMP
all	0.00	0.00	0.00	0.00	0.00	6	6	CMP
global	0.34	0.22	0.39	0.30	0.24	6	7	CMP
any	0.34	0.22	0.39	0.30	0.24	6	7	CMP
all	0.00	0.00	0.00	0.00	0.00	6	7	CMP
global	0.88	0.31	0.91	0.43	0.35	6	8	CMP
any	0.88	0.31	0.91	0.43	0.35	6	8	CMP
all	0.00	0.00	0.00	0.00	0.00	6	8	CMP
global	0.21	0.31	0.31	0.37	0.32	6	9	CMP
any	0.21	0.31	0.31	0.37	0.32	6	9	CMP
all	0.00	0.00	0.00	0.00	0.00	6	9	CMP
global	0.30	0.56	0.46	0.59	0.55	6	10	CMP
any	0.30	0.56	0.46	0.59	0.55	6	10	CMP
all	0.00	0.00	0.00	0.00	0.00	6	10	CMP
global	0.17	0.18	0.18	0.28	0.21	6	11	NB
any	0.17	0.18	0.18	0.28	0.21	6	11	NB
all	0.00	0.00	0.00	0.00	0.00	6	11	NB
global	0.24	0.14	0.24	0.25	0.16	6	12	NB
any	0.24	0.14	0.24	0.25	0.16	6	12	NB
all	0.00	0.00	0.00	0.00	0.00	6	12	NB
global	0.13	0.21	0.23	0.30	0.21	6	13	NB
any	0.13	0.21	0.23	0.30	0.21	6	13	NB
all	0.00	0.00	0.00	0.00	0.00	6	13	NB
global	0.19	0.24	0.28	0.33	0.25	6	14	NB
any	0.19	0.24	0.28	0.33	0.25	6	14	NB
all	0.00	0.00	0.00	0.00	0.00	6	14	NB

Table S4 Power-types for contrast Tukey, $\tilde{\lambda}_1 = \lambda, \tilde{\lambda}_2 = \lambda + 1.5, \tilde{\lambda}_3 = \lambda + 2.5, \tilde{\lambda}_4 = \lambda + 3$ and $\lambda = 6$, all null hypotheses are false.

2 Simulation results for three groups

Table S5 Overview of simulated designs with $\mathbf{n_1} = (6, 6, 6)'$, $\mathbf{n_2} = (6, 10, 16)'$ and $m \in \{0, 2, 4, \ldots, 24\}$.

Setting	Distrib.	Sample sizes	Dispersion	Dispersion parameter	Interpretation
1	POI	$\mathbf{n} = \mathbf{n_1} + m$	equidispersed	-	Balanced homosc.
2	POI	$\mathbf{n} = \mathbf{n_2} + m$	equidispersed	-	Unbalanced homosc.
3	CMP	$\mathbf{n} = \mathbf{n_1} + m$	overdispersed	$\boldsymbol{\nu} = (0.5, 0.5, 0.5)'$	Balanced homosc.
4	CMP	$\mathbf{n} = \mathbf{n_1} + m$	underdispersed	$\boldsymbol{\nu} = (2, 2, 2)'$	Balanced homosc.
5	CMP	$\mathbf{n} = \mathbf{n_1} + m$	overdispersed	$\boldsymbol{\nu} = (0.2, 0.5, 0.5)'$	Balanced heterosc.
6	CMP	$\mathbf{n} = \mathbf{n_1} + m$	over- & underd.	$\boldsymbol{\nu} = (0.2, 0.5, 2)'$	Balanced heterosc.
7	CMP	$\mathbf{n} = \mathbf{n_2} + m$	overdispersed	$\boldsymbol{\nu} = (0.5, 0.5, 0.2)'$	Positive pairing
8	CMP	$\mathbf{n} = \mathbf{n_2} + m$	over- & underd.	$\boldsymbol{\nu} = (2, 0.5, 0.2)'$	Positive pairing
9	CMP	$\mathbf{n} = \mathbf{n_2} + m$	overdispersed	$\boldsymbol{\nu} = (0.2, 0.5, 0.5)'$	Negative pairing
10	CMP	$\mathbf{n} = \mathbf{n_2} + m$	over- & underd.	$\boldsymbol{\nu} = (0.2, 0.5, 2)'$	Negative pairing
11	NB	$\mathbf{n} = \mathbf{n_1} + m$	overdispersed	**size** $= (3, 3, 3)'$	Balanced homosc.
12	NB	$\mathbf{n} = \mathbf{n_2} + m$	overdispersed	**size** $= (5, 3, 2)'$	Positive pairing
13	NB	$\mathbf{n} = \mathbf{n_2} + m$	overdispersed	**size** $= (0.75, 2, 3)'$	Negative pairing
14	NB	$\mathbf{n} = \mathbf{n_2} + m$	overdispersed	**size** $= (2, 3, 5)'$	Negative pairing

Rate parameter $\lambda_i \in \{1, 6, 10\}$ with $i \in \{1, 2, 3\}$, $\mathbf{n_j} + m$ means that each component of $\mathbf{n_j}$ increases by m.

Type I error

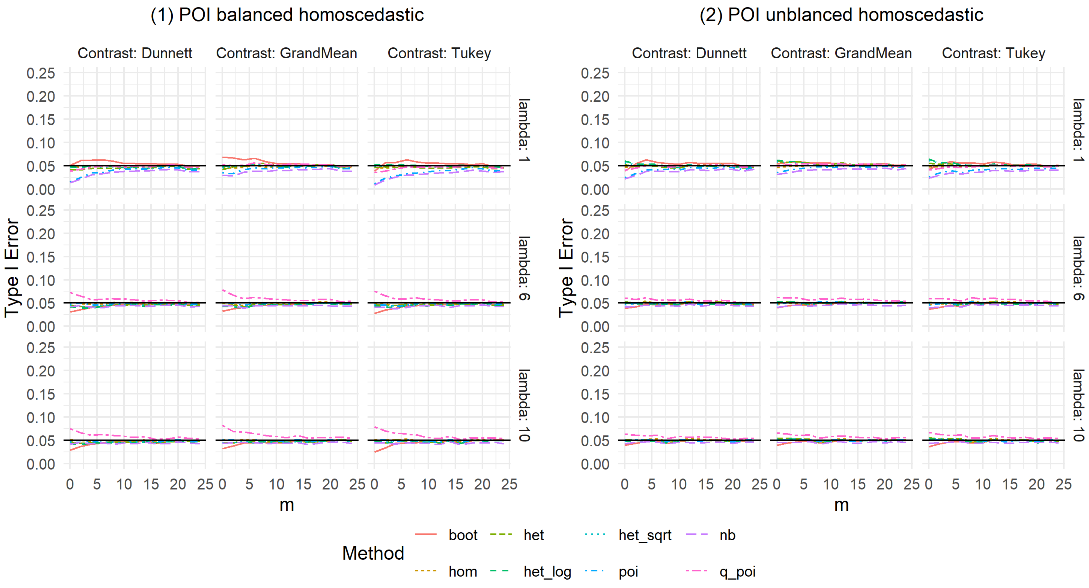

Figure S1 Type I error simulation for Poisson distributed data.

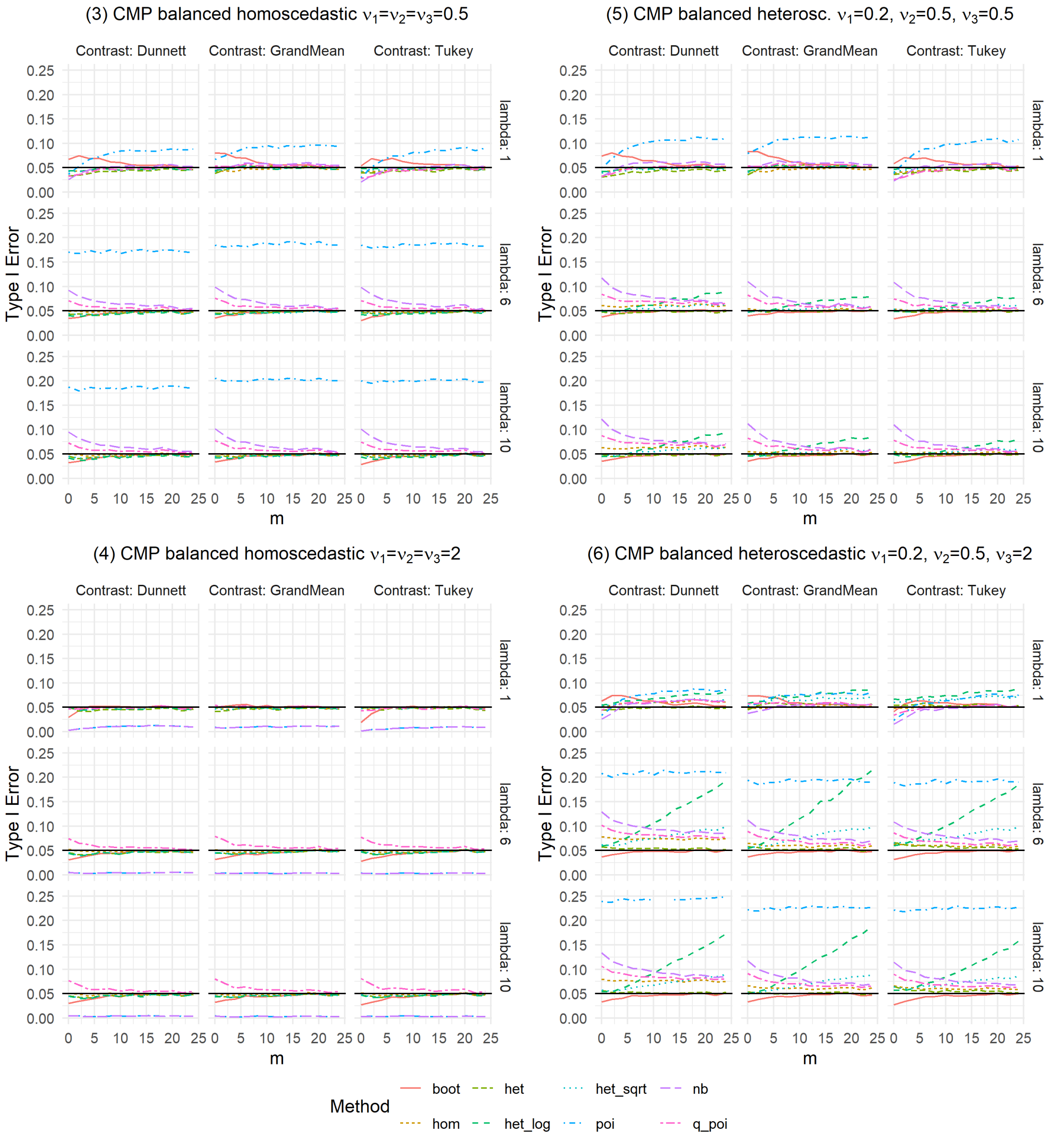

Figure S2 Type I error simulation for balanced settings of Conway-Maxwell Poisson distributed data with overdispersion ($\nu \in \{0.2, 0.5\}$), underdispersion ($\nu = 2$) and combinations of both.

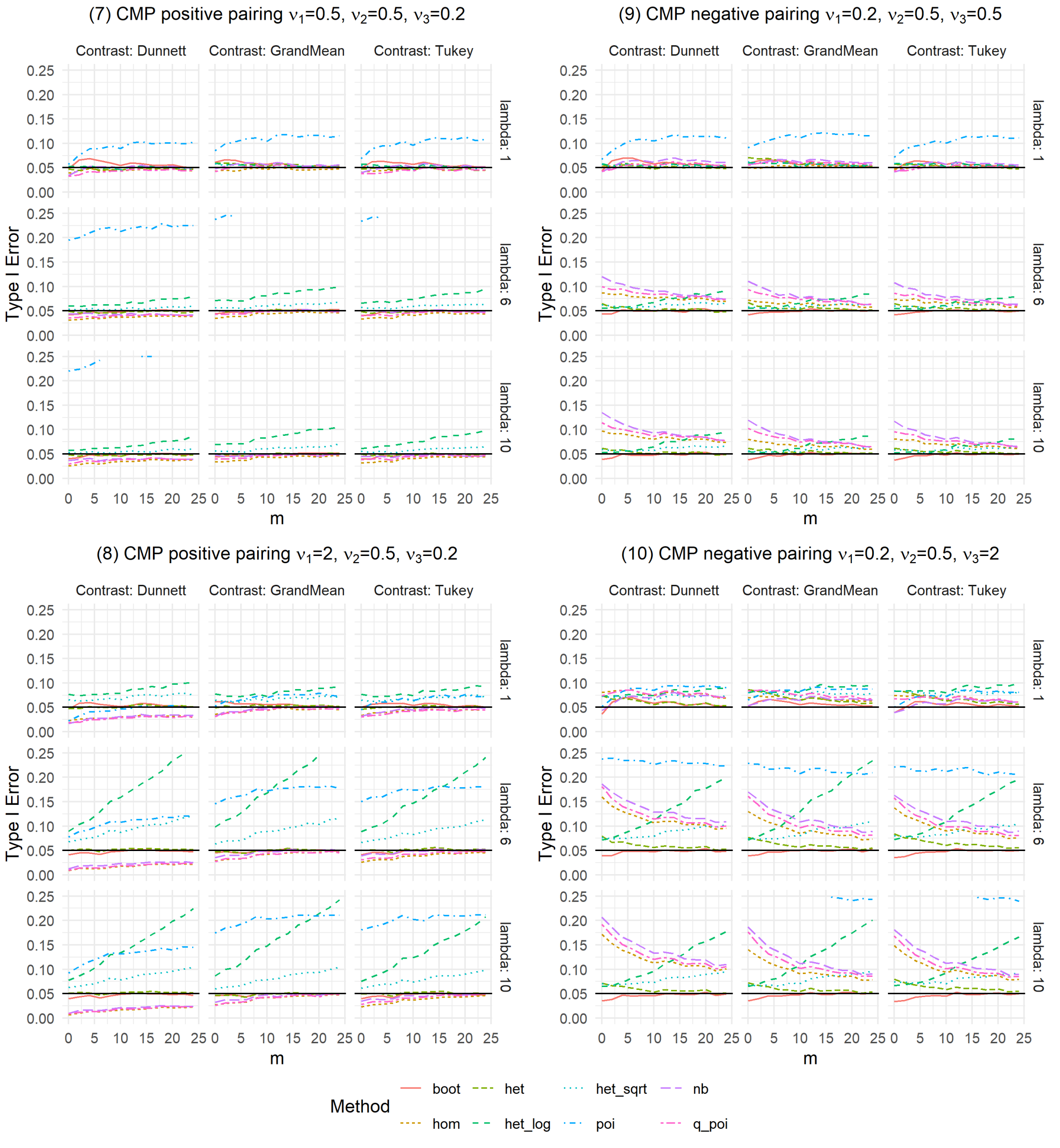

Figure S3 Type I error simulation for unbalanced settings of Conway-Maxwell Poisson distributed data with overdispersion ($\nu \in \{0.2, 0.5\}$), underdispersion ($\nu = 2$) and combinations of both.

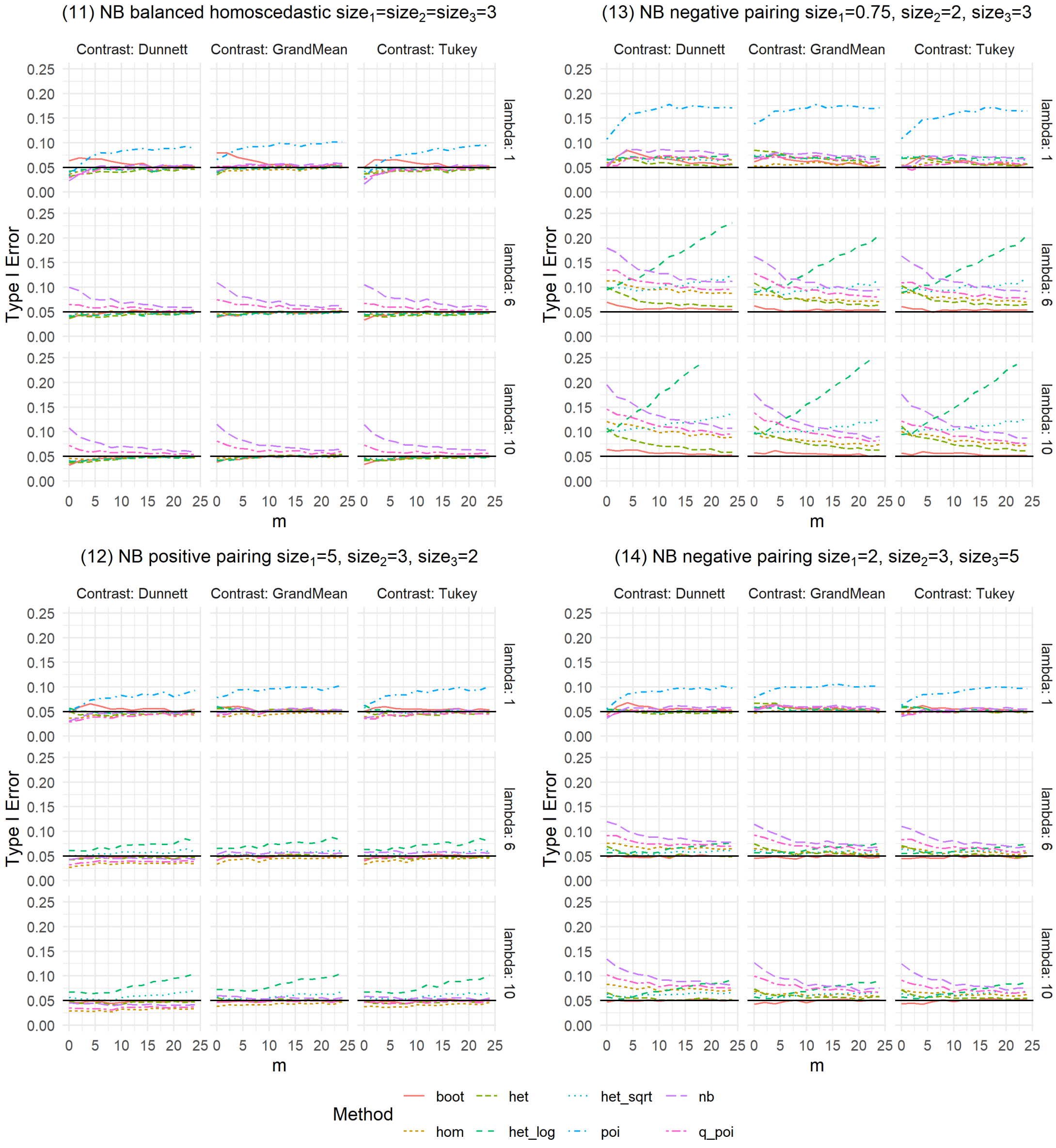

Figure S4 Type I error simulation for Negative Binomial distributed data.

Power

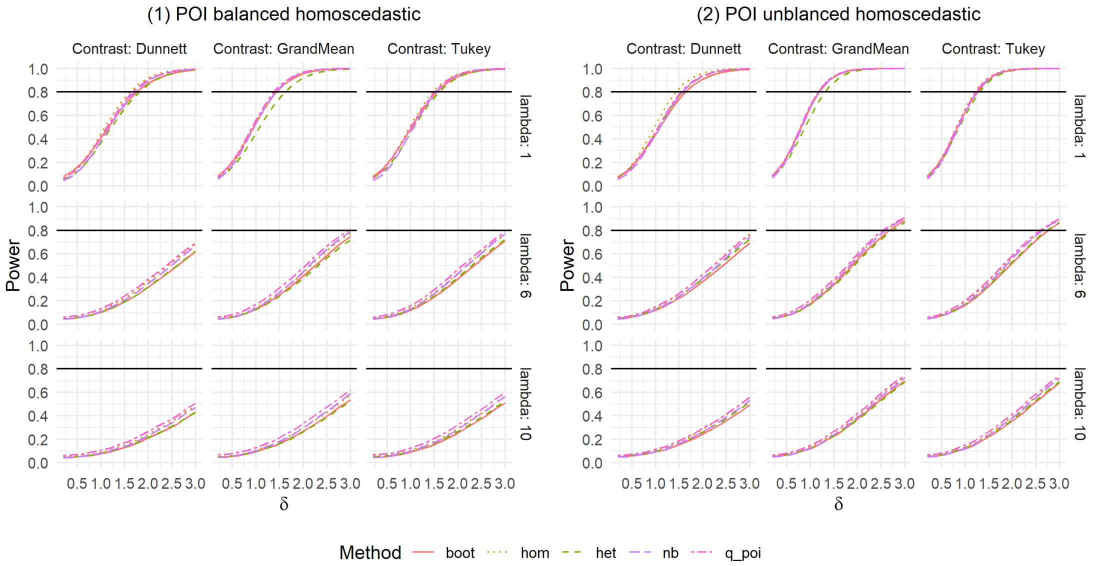

Figure S5 Power simulation for Poisson distributed data.

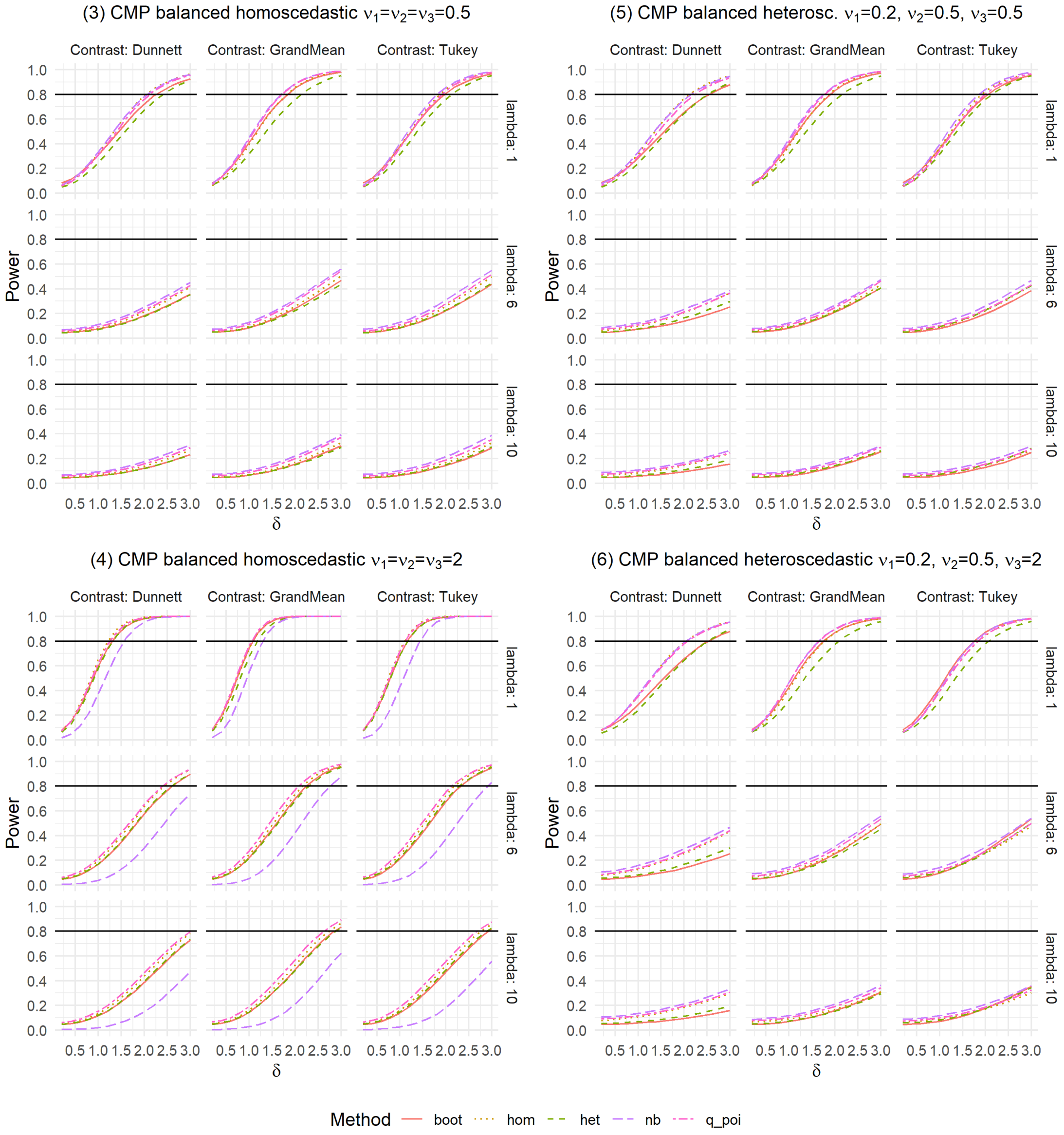

Figure S6 Power simulation for Conway-Maxwell Poisson distributed data with balanced settings.

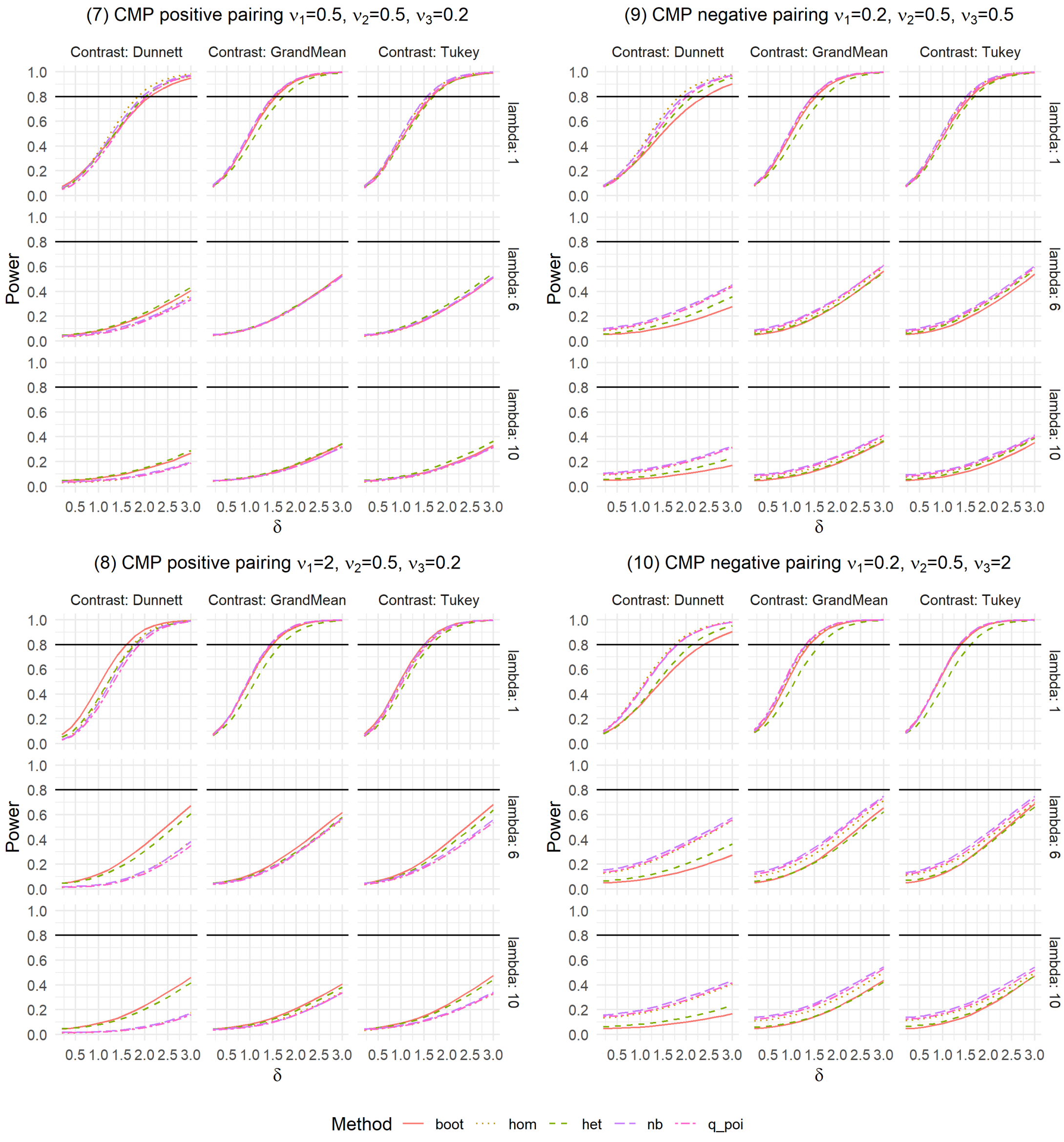

Figure S7 Power simulation for Conway-Maxwell Poisson distributed data with positive and negative pairing.

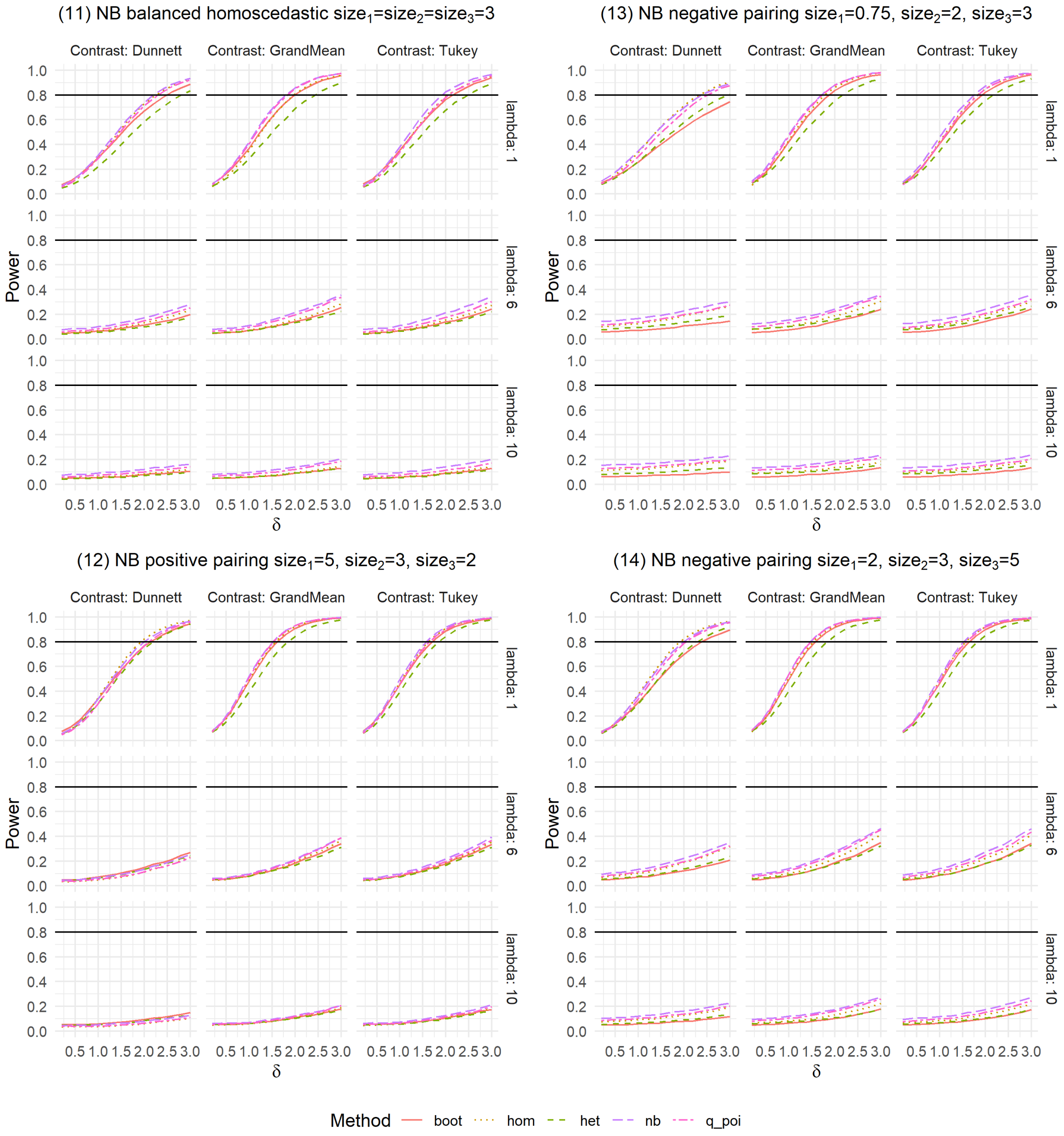

Figure S8 Power simulation for Negative Binomial distributed data.
